# Magnetic Monopole‐Like Behavior in Superparamagnetic Nanoparticle Coated With Chiral Molecules

**DOI:** 10.1002/smll.202406631

**Published:** 2024-08-29

**Authors:** Qirong Zhu, Sidney R. Cohen, Olga Brontvein, Jonas Fransson, Ron Naaman

**Affiliations:** ^1^ Department of Chemical and Biological Physics Weizmann Institute of Science Rehovot 76100 Israel; ^2^ Department of Chemical Research Support Weizmann Institute of Science Rehovot 76100 Israel; ^3^ Department of Physics and Astronomy Uppsala University Box 516 Uppsala 75120 Sweden

**Keywords:** atomic force microscopy, chirality, magnetic monopole, superparamagnetic iron oxide nanoparticles

## Abstract

Superparamagnetic iron oxide nanoparticles (SPIONs) have attracted wide attention due to their promising applications in biomedicine, chemical catalysis, and magnetic memory devices. In this work, the force is measured between a single SPION coated with chiral molecules and a ferromagnetic substrate by atomic force microscopy (AFM), with the substrate magnetized either toward or away from the approaching AFM tip. The force between the coated SPION and the magnetic substrate depends on the handedness of the molecules adsorbed on the SPION and on the direction of the magnetization of the substrate. By inserting nm‐scale spacing layers between the coated SPION and the magnetic substrate it is shown that the SPION has a short‐range magnetic monopole‐like magnetic field. A theoretical framework for the nature of this field is provided.

## Graphical abstract

Magnetic monopole‐like phenomenon is observed for a superparamagnetic iron oxide nanoparticle (SPION) coated with chiral molecules. Combining force spectroscopy and calculations, this study reveals that the magnetic moment of the SPION behaves like a monopole at close proximity to the particle, at distance of less than 10 nm, the nanoparticle diameter.

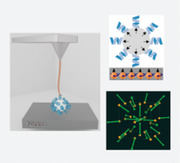

## 1 Introduction

Magnetism is always associated with a dipolar field, in contrast to electric charge, which is a monopole that can behave as isolated south or north magnetic poles. Following Dirac's theory of magnetic monopoles,^[^ ^1^ ^]^ there were efforts to detect magnetic monopoles.^[^ ^2^ ^]^ Indeed, magnetic behavior analogous to monopoles was observed in various exotic systems, such as spin ices and skyrmion crystals in chiral magnets.^[^ ^3^, ^4^, ^5^, ^6^, ^7^ ^]^ A monopole‐like magnetic field was also created by a spinor Bose–Einstein condensate^[^ ^8^ ^]^ and by topological surface states.^[^ ^9^ ^]^ In all the cases described in the past, the monopole‐like field was created, however it could not be transferred or applied at will. Here, we present hybrid structures made from superparamagnetic iron oxide nanoparticles coated with a monolayer of chiral molecules. We show experimentally and by calculations that each such nanoparticle behaves as a magnetic monopole in the near field while the magnetic field is zero at the far field. The special magnetic properties result from the chiral‐induced spin selectivity (CISS) effect^[^ ^10^, ^11^, ^12^, ^13^ ^]^ that causes the polarization of the spin of the outer layer of the iron oxide, so that all the spins are aligned outward from or inward toward the nanoparticle, depending on the handedness of the adsorbed chiral molecules. Hence, these particles show magnetic monopole‐like properties in the near field and can be used in technologies such as energy storage.^[^ ^14^ ^]^

**Figure 1:**
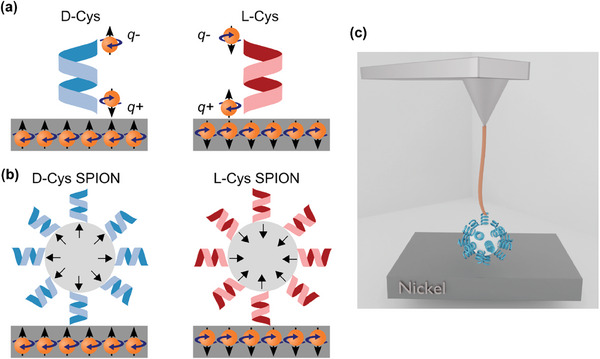
a) The charge and spin distribution upon the approach of a chiral molecule to the ferromagnetic surface. Upon approach an induced electric dipole moment is formed (q+ and q‐) accompanied by spin polarization. The handedness of the molecule determines which spin is associated with which electric pole. b) For the molecule to bind to the superparamagnetic particles, the spins in the outer layers of the particles are aligned to be antiparallel to the spin on the molecular binding site. As a result, the spin alignment on the surface of the nanoparticles is defined by the handedness of the molecules. c) A scheme of the SPION attached to the AFM tip via the polyethylene glycol (PEG) linker.

It was found that electron motion through chiral molecules is spin dependent, consistent with the CISS effect.^[^ ^15^ ^]^ This phenomenon results in spin polarization and associated charge polarization in chiral systems. Hence, when chiral molecules approach a substrate and charge is reorganized in the molecule, the difference in electrochemical potential between the molecule and the substrate result in a (partial) spin polarization that is associated with each electric pole (see **Figure** **1**a). Which spin is associated with which pole depends on the handedness of the molecule.^[^ ^16^ ^]^

When chiral molecules are chemically adsorbed on ferromagnetic substrates, the spin polarization in their head group, which binds to the substrate, can induce alignment of the spins in the first few layers below the surface (Figure 1b).^[^ ^17^ ^]^ The effect of adsorption of chiral molecules on superparamagnetic nanoparticles was investigated and it was found that the nanoparticles behave as ferromagnets.^[^ ^18^, ^19^ ^]^ However, the exact distribution of the magnetic moment of these particles was not determined.

In the present work, we chemisorbed D or L cysteine (D‐Cys or L‐Cys) molecules on 10 nm diameter superparamagnetic iron oxide nanoparticles (SPIONs). A single SPION was attached to the tip of an atomic force microscope (AFM) through a polyethylene glycol (PEG) linker, to eliminate the non‐specific interaction of the tip on the force between the SPION and the substrate (Figure 1c). The ferromagnetic properties of the substrate are due to a nickel (Ni) film which was magnetized by flipping the direction of the permanent magnet, thus aligning the spin. The Ni film was coated by a thin titanium (Ti) film to inhibit chemical interaction between the thiol group on the cysteine and the Ni. The thickness of the Ti film as well as the use of an aluminum oxide spacer layer served as controls to modulate the influence of the magnetic field. The system was characterized in detail, as described in the supporting information (Figures S1–S16, Supporting Information). The SPIONs were brought into contact with the substrate and the force required to detach them from the ferromagnetic substrate^[^ ^20^, ^21^, ^22^ ^]^ was measured as a function of the magnetization direction of the substrate (Figures S17–S21, Supporting Information).

There are various interactions that determine the force required to detach the SPION from the surface. Among them are electrostatic and van der Waals interactions. The van der Waals force is unaffected by the electrolyte concentration and pH of the buffer solution, while the electrostatic force depends on these variables.^[^ ^23^, ^24^ ^]^ The electrostatic interaction between AFM tip and substrate can be minimized by operating in electrolytes, e.g., phosphate‐buffered saline (PBS buffer).^[^ ^25^ ^]^ The electrostatic force decreases when the concentration of the electrolytes increases.^[^ ^26^ ^]^ Therefore, the effective pulling force is dominated by the van der Waals force, the (short‐range) spin exchange interaction, and the magnetic interaction. The way to measure the contribution of the spin exchange and the magnetic force is to flip the magnetization direction of the ferromagnetic substrate. Changing the direction of the magnetic field does not affect the van der Waals interaction.

As was shown before, chiral molecules interact with ferromagnetic surfaces through spin exchange interactions. This interaction depends on the handedness of the chiral molecules and on the direction of the magnetic field.^[^ ^27^ ^]^ It does not depend on the orientation of the SPION relative to the ferromagnetic surface. The same is true if the SPION behaves as a monopole, in which case the interaction will flip between attractive and repulsive when the direction of the magnet moment of the substrate is reversed. Hence, for both spin exchange and magnetic monopole‐like interactions, for one direction of the substrate magnetic moment, the absolute force measured will be larger than the force without the magnetic field, while for the other direction it will be smaller. The way to distinguish between the exchange and magnetic forces is by their range. Spin exchange interaction decays exponentially with the distance between the ferromagnet and the particle and is typically effective only in the sub‐nanometer range, while the magnetic field effect decays as a power law and is expected to decay on the scale of the size of the SPION, i.e., 10 nm.

## 2 Results

**Figure 2:**
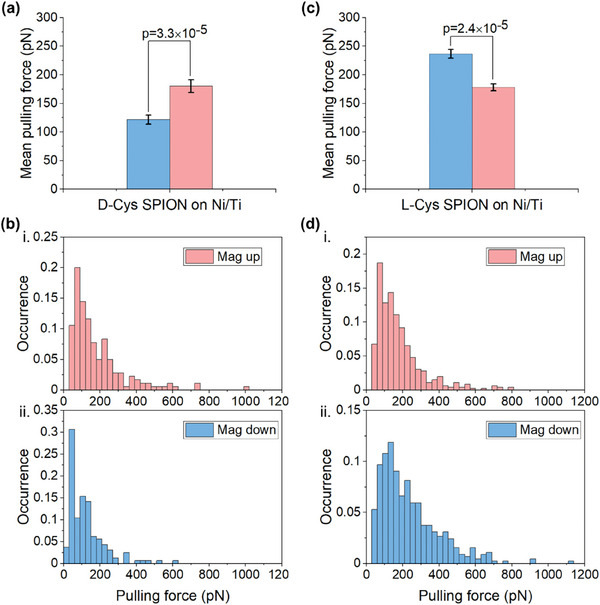
AFM‐based force spectroscopy measurements of D/L‐Cys SPION interacting with Ni/Ti (120/2.9 nm) substrates in PBS buffer. a,b) MPF and histograms of the pulling force between the D‐Cys SPION and the Ni/Ti substrate under i) up magnetization (mag up) and ii) down magnetization (mag down). c,d) MPF and histograms of the pulling force between the L‐Cys SPION and the Ni/Ti substrate. The pulling force was recorded at a retracting speed of 400 nm s^−1^. Error bars represent the standard error of the mean. The p‐value shown represents the 2‐sample *t*‐test: for both enantiomers the null hypothesis (that MPF is not significantly different) has probability of well under 10^−4^. Results shown used a single tip for each SPION chirality.

In the first measurement, a thin Ti layer (2.9 ± 0.1 nm) was evaporated onto the Ni surface to avoid chemical interaction between the thiol group on the cysteine and the Ni and to minimize the spin exchange interaction. After modifying the AFM tip, the force measurement was performed in PBS (pH 7.2) with a retract speed of 0.4 µm s^−1^. **Figure** **2** shows the mean pulling force (MPF) and the histograms for the force measured between D/L‐Cys SPION and the Si/SiO_2_/Ti(10 nm)/Ni(120 nm)/Ti(2.9 ± 0.1 nm) substrate, when the Ni is either magnetized toward the AFM tip (mag up) or away from it (mag down). For D‐Cys SPION interacting with Ni/Ti, the MPF values for mag up and mag down are 180.2 ± 11.1 pN and 121.8 ± 7.8 pN, respectively (Figure 2a). Figure 2b presents the corresponding histograms of pulling force for mag up and mag down for D‐Cys SPION (see more details in Figure S19, Supporting Information). The MPF of L‐Cys SPION for mag up and mag down are 178.3 ± 6.0 and 236.6 ± 7.6 pN, respectively (Figure 2c). The corresponding histograms are shown in Figure 2d. The force difference in MPF for both enantiomers, comparing magnet pointing up and down is 58 pN. Specifically, applying a 2‐sample *t*‐test, the probability (p‐value) that the up/down magnetizations are equivalent is 3.3 × 10^−5^ for D‐Cys SPION and 2.4 × 10^−5^ for L‐Cys SPION. We can therefore conclude that the pull‐off force difference between up and down magnetizations is significantly different.

The results in Figure 2 clearly demonstrate that the magnetic properties of the SPIONs are defined by the handedness of the chiral molecules and are not affected by the magnetization of the substrate. This is consistent with former studies that indicate that the interaction of chiral molecules with magnetic susbtrates is on the order of hundreds meV, much larger than the influence of external magnetic field.^[^ ^17^ ^]^

**Figure 3:**
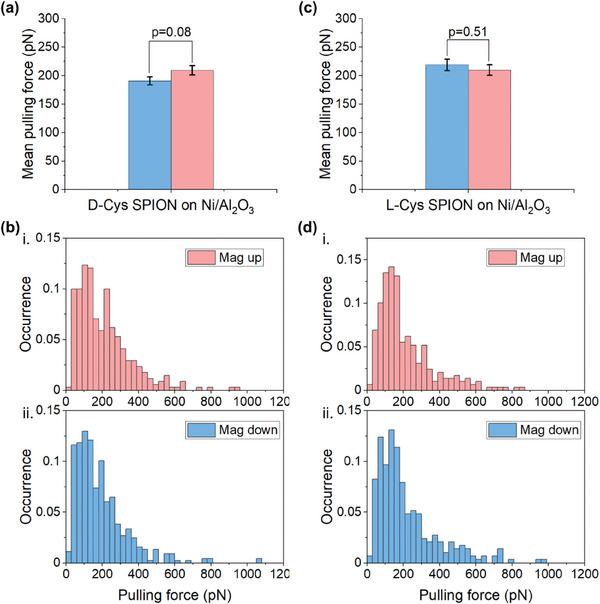
AFM‐based force spectroscopy measurements of D/L‐Cys SPION on the Ni/Al_2_O_3_ substrates in PBS buffer. a,b) MPF and histograms of the pulling force between the D‐Cys SPION and the Ni/Al_2_O_3_ (120/8.9 nm) substrate under i) up magnetization (mag up) and ii) down magnetization (mag down). c,d) MPF and histograms of the pulling force between the L‐Cys SPION and the Ni/Al_2_O_3_ substrate. The pulling force was recorded at a retracting speed of 400 nm s^−1^. Error bars represent the standard error of the mean. The Al_2_O_3_ film is insulating which partially blocks the charge and spin injection from the substrate. The calculated p values are >0.05 indicating that there is no significant difference between the up/down directions. Results shown used a single tip for each SPION chirality.

As a control experiment, we inhibit completely the spin‐exchange interaction by evaporating an insulating layer (aluminum oxide layer with a thickness of 8.9 ± 0.2 nm) onto the Ni surface. **Figure** **3** shows the MPF between D/L‐Cys SPION and the Si/SiO_2_/Ti(10 nm)/Ni(120 nm)/Al_2_O_3_(8.9 nm) substrate for the mag up and mag down directions. For D‐Cys SPION, the MPF values for mag up and mag down are 209.2 ± 8.1 and 190.7 ± 6.9 pN, respectively (shown in Figure 3a). Figure 3b presents the corresponding histograms (see more details in Figure S20, Supporting Information). For L‐Cys SPION, the MPF for mag up and mag down are very similar 209.9 ± 9.1 and 218.9 ± 10.0 pN, respectively (Figure 3c,d; Figure S20, Supporting Information). Here, the p‐values for D‐Cys SPION and L‐Cys SPION are 0.08 and 0.51, respectively. Since the p‐values are more than 0.05, there is no significant difference between the up/down magnetization force for D/L‐Cys SPION on the Ni/Al_2_O_3_ substrate.

**Figure 4:**
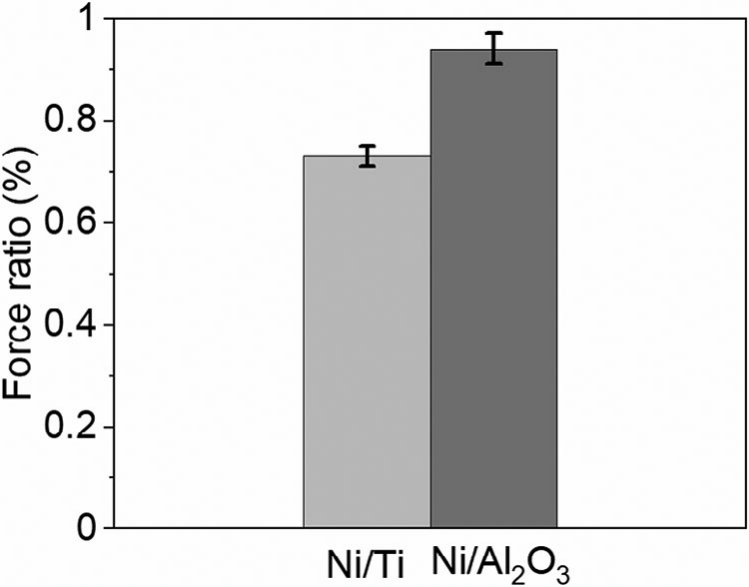
The ratio of averaged MPF between up and down magnetization: 73 ± 4% for the Ni/Ti substrate (4 tips) and 94 ± 6% for the Ni/Al_2_O_3_ substrate (5 tips). The error bar is the standard deviation of the mean.

For D‐ versus L‐Cys SPIONs, at this larger distance from the Ni surface, these results indicate a weak or no significant dependence on the direction of magnetization of the substrate. The force difference of MPF on Ni/Al_2_O_3_ surface, between the different magnetic directions, is 15.4 ± 10.0 pN, about one‐third the value for the measurements made on the Ni surface with 2.9 nm‐thick spacer, 51.6 ± 7.9 pN (see Figure S21, Supporting Information). Figures 2 and 3 show representative results from 2000 pull‐off curves, using a single tip for each enantiomer. These experiments were repeated with different tips and newly prepared surfaces to verify their repeatability. Since each tip has unique SPION size, which influences the absolute force, we represent for each such experiment the ratio of up/down MPF for L chirality and down/up for D chirality. This allows combining the results of all similar tip/sample sets. The results are shown in **Figure** **4** for Ni/Ti and Ni/Al_2_O_3_. The ratio is 73 ± 4% for Ni/Ti and 94 ± 6% for Ni/Al_2_O_3_. This indicates that while for the Ni/Ti substrate there is some magnetic field effect, for the thicker Ni/Al_2_O_3_ there is no significant difference. Thus the combined data from several different experiments is consistent with the data shown in Figures 2 and 3. The supression of the magnetic field effect relates, most probably, to the thickness of the Al_2_O_3_ spacer layer, which is of the order of the diameter of the SPION and hence results in reduction of the magnetic interaction. To verify that the data we collected are due to the chiral SPION, a control experiment was performed measuring the force between the PEG linker modified AFM tip (no SPION) and the Al_2_O_3_ coated Ni surface (Figure S22, Supporting Information). Without the chiral SPION, the MPF of mag up and mag down are identical, within experimental uncertainty, 48.4 ± 2.4 pN and 50.3 ± 2.0 pN, respectively. As a further control, we performed force measurements between the SPION coated with achiral molecules (3‐mercaptopropionic acid, abbreviated as 3‐MPA) and the Ni/Ti (120/2.9 nm) surface. The results are displayed in Figure S23 (Supporting Information). For the achiral SPIONs, the measured forces are 223.6 ± 17.9 pN for up, and 224.1 ± 7.4 pN for downward magnetic orientation. These nearly identical forces, under opposite magnetic fields, confirm that the chirality of the attached molecules is essential for achieving spin alignment in the SPION, as indicated by the significant force difference, for these SPIONS, under opposite magnetic fields.

In another control experiment, a thicker Ti layer (9.4 ± 0.2 nm) was evaporated on the Ni surface and the force between L‐Cys SPION and the magnetized substrate was measured (Figure S24, Supporting Information). The MPFs for mag up and mag down are 169.9 ± 10.4 pN and 171.9 ± 9.6 pN, respectively. Hence increasing of the thickness of the diamagnetic Ti layer, results in eliminating the effect of the magnetic interaction.

## 3 Discussion

Previous work showed that SPIONs, coated with a chiral molecule have no net magnetization because the spherical symmetry is not broken.^[^ ^18^ ^]^ This observation is confirmed by our superconducting quantum interference device (SQUID) measurements of densely packed D‐Cys and L‐Cys‐covered SPIONs (Figure S25, Supporting Information). In the AFM measurements, the ferromagnetic substrate breaks the spherical symmetry, and for distances smaller than the nanoparticle diameter, the magnetic layer interacts only with the part of the SPION in proximity to it (see Figure S26, Supporting Information). Therefore, the local magnetic field interacts directly with the surface of the SPION. It is important to appreciate that the distance between the SPION and the magnetic layer defines the mechanism of the interaction. If the SPIONs are in direct contact with the Ni layer, then the spin exchange interaction and magnetic forces are sensitive to the direction of magnetization of the Ni. When a diamagnetic layer is deposited between the Ni and the SPIONs, the effect of the spin exchange interaction is diminished. This is observed for the diamagnetic Ti layer (9.4 ± 0.2 nm) as well as the Al_2_O_3_ layer (8.9 ± 0.2 nm). At this larger distance, the effect of magnetic interaction is diminished since the substrate interacts more uniformly over the entire surface of the spherical nanoparticle resulting in a dramatic decrease in the net field.

It is useful to visualize the arrangement of the magnetic dipoles within the SPIONs so as to form the monopole‐like a magnetic moment in proximity to the nanoparticles.

**Figure 5:**
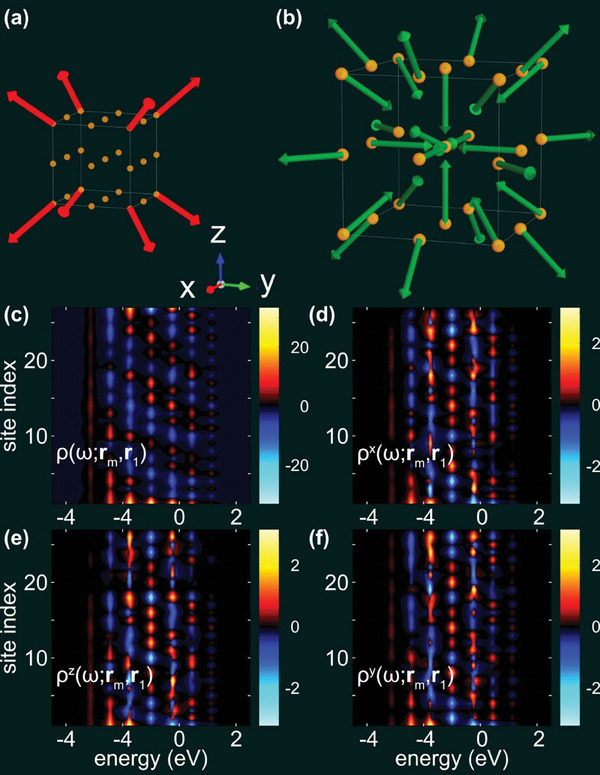
Calculations of magnetic properties. a) Real space configuration of the lattice with external magnetic moments (red arrows) included. b) Induced magnetic moments (green arrow). c–f) Correlation functions (c) ρ(ω; *r_m_*,*r*_1_) and d–f) ρ^α^(ω; *r_m_*,*r*_1_), α = *x*, *y*, *z*, as function of the energy (ω) and sites (*m* – vertical axis). The color code is in scale between the panels and indicate negative (blue scale) and positive (yellow scale) values of the interaction density. In the calculations we have used values ε_*m*_ − µ  =   − 1, for all *m*, *t* = 0.5, *J* = 0.2, and *v*_J_ = 0.005 [units: eV], where µ is the overall chemical potential, and the temperature *T* = 300 K.

We model the induced magnetization in three steps, of which the first consists of calculating the effective electronic structure of the nanoparticle comprising *N* sites in cubic configuration, see Figure 5a on top of which chiral molecules are adsorbed. The adsorbed chiral molecules are charge‐polarized and this charge polarization is accompanied by spin polarization. These phenomena were discussed before for chiral molecules adsorbed on no ferromagnetic substrates.^[^ ^16^, ^28^ ^]^ In the second step, this electronic structure is used for calculating the electronically mediated spin‐spin interactions in the effective spin model of the nanoparticle. In the third and final step, the spin model is self‐consistently solved in order to determine the local and global magnetic structure.

The first step is based on a tight‐binding model $H_{0}=\sum_{m}\varepsilon_{m}\psi_{m}^{†}\psi_{m}-t\sum_{\langle mn\rangle }\psi_{m}^{†}\psi_{n}$ for the cubic structure, where ψ_*m*_ =  (ψ_*m*↑_ψ_*m*↓_)^*t*^ is the spinor for electrons at site *m* and energy ε_*m*_. Electrons can move to its nearest neighbors (indicated by 〈*mn*〉) with rate *t*. Local external spin moments ***S**_i_* are located at the eight corners of the cube, labelled by *i*. These spins are associated with the adsorbed chiral molecules. Their moments are pointing outward and interact with electrons at the adjacent sites via direct exchange *J*, expressed in the form $H_{\mathrm{int}}=-J\sum_{i}S_{i}\cdot \psi_{i}^{†}\sigma \psi_{i}$, where ***σ*** is the vector of Pauli matrices. The configuration of the initial set‐up is illustrated in **Figure** **5**a, where brown balls represent the (*N*  =  27) sites in the lattice and red arrows represent the attached local spin moments.

The goal is to obtain the induced spin configuration of the spin moments ***s**_m_* associated with sites *m* in the lattice. For generality, we assume that the spins interact with one another through isotropic and anisotropic exchange, where the latter is decomposed into symmetric and anti‐symmetric contributions. Mathematically, this is expressed as the Hamiltonian^[^ ^29^, ^30^ ^]^

(1) $$H_{S}=\sum_{mn}\;s_{m}\cdot \left(J_{mn}s_{n}+D_{mn}\times s_{n}+II_{mn}\cdot s\right)$$

where *J_mn_*, ***D**_nm_*, and Π_*mn*_ denote the isotropic (scalar), anti‐symmetric (three component vector), and symmetric (3 × 3 matrix) anisotropic exchange interactions, respectively. Here, it should be stressed that we include interactions between all sites. The interaction coefficients are calculated using the results from the electronic structure calculation in the first step, assuming that the electrons and spin moment at site *m* interact via direct exchange *v*_J_ in the same form as $H_{int}$. Specifically, the calculated electronic structure is expressed in terms of the matrix Green functions ***G*** (ω)  =  {***G**_mn_*}_*mn*_. Furthermore, each matrix entry ***G**_mn_* is a 2 × 2‐matrix Green function in spin 1/2 space connecting sites *m* and *n*. Therefore, we can write $G_{mn}=G_{mn}^{\left(0\right)}\;\sigma^{0}+G_{mn}^{\left(1\right)}\cdot \sigma$, where $G_{mn}^{\left(0\right)}$ (scalar) and $G_{mn}^{\left(1\right)}=\;{\left\{G_{mn}^{\left(\alpha \right)}\right\}}_{\alpha \;=\;x,y,z}$ (three component vector) represent the charge and spin properties of the lattice. An example of these Green function components is plotted in Figure 5c–f, showing the correlation functions $\rho \left(\omega ;r_{m},r_{1}\right)\;=\;-ImG_{m1}^{r,(0)}\left(\omega \right)/\pi$ and $\rho^{\left(\alpha \right)}\left(\omega ;r_{m},r_{1}\right)\;=\;-ImG_{m1}^{r,(\alpha )}\left(\omega \right)/\pi$, for m=1,···,N. The contour plots clearly illustrate that there is a non‐negligible coupling between sites m=1,···,N and *n*  =  1.

Using this representation, we calculate the exchange coefficients using the formulas derived in Refs. [18, 28], for instance,

(2) Jmn=−vJ2π∫fωImGmnr,0ωGnmr,0ω−Gmnr,1ω·Gnmr,1ωdω

where *f*(ω) is the Fermi‐Dirac distribution function. The expression explicitly underlines the fact that not only the charge ($G_{mn}^{\left(0\right)}$) but also the spin ($G_{mn}^{\left(1\right)}$) contributes to the total indirect exchange interaction between the spin moments. Therefore, both the symmetric and anti‐symmetric anisotropic interactions are non‐negligible, which is crucial in order to obtain the result in the third step. It should also be noticed that the interaction coefficients are multiplied by the decay factor $R_{0}/\left({|r_{m}-r_{n}|}^{2}+R_{0}^{2}\right)$ to account for the distances between the sites.

In the third step, we calculate the spin configuration of the model in Equation (1) self‐consistently in the random phase approximation (Tyablikov's decoupling).^[^ ^31^ ^]^ The calculated spin configuration corresponding the electronic structure plotted in Figure 5c–f is illustrated in Figure 5b, where the green arrows indicate the spatial orientation of the spin moments at each site. The length and direction of the arrow at site *m* is proportional to the expectation value $\left\langle s_{m}\right\rangle \;=\;\left\langle s_{m}^{x},s_{m}^{y},s_{m}^{z}\right\rangle$, such that the arrows are plotted in scale with each other. The visualization of the final spin structure shows that the spins point in directions, which compensate the presence of the external spin moments. In particular, the spins are arranged such that the sum of the local moments vanishes. The finite spin moment at the center of the cube in Figure 5b results from the odd number of spins and the fact that | ***s**_m_* | is constant.

Hence, the model visualizes the possibility to have a nano particle with a “monopole like” magnetic moment, with all the moments pointing outward. Within the particle the magnetic moments cancel each other to a large extent.

## 4 Conclusion

In this work, we used chiral molecules with different handedness to modify the SPIONs and then attach them to an AFM tip. The handedness of the adsorbed chiral molecules defines the spin orientation near the surface of the SPION^[^ ^17^ ^]^ and hence the direction of the short‐range magnetic field. Using force‐microscopy to probe the local interaction force between the chiral SPION and the magnetically aligned ferromagnetic substrate, we conclude that the magnetic moment of the SPION behaves as a monopole at close proximity to the particle. By coating the magnetic substrate with different thicknesses of a diamagnetic spacer layer we show that the interaction is indeed magnetic and that it decays sharply as the spacing thickness approaches the nanoparticle diameter.

## 5 Experimental Section

### Materials

D‐cysteine (99%), L‐cysteine (BioUltra, 98.5%), 3‐mercaptopropionic acid (≥99%), Tris(2‐carboxyethyl)phosphine hydrochloride (TCEP) and magnetic nanoparticle solution of iron oxide (II, III) (10 nm avg. part. size, 5 mg mL^−1^ in H_2_O) were purchased from Sigma‐Aldrich Co. LLC. Maleimide‐poly(ethylene glycol)‐silane (MAL‐PEG‐Silane, 5k) was bought from Creative PEGworks. The AFM probes (Bruker SNL‐10, cantilever D, nominal tip radius = 2 nm, nominal spring constant = 0.06 N/m) were made from silicon nitride. Ultra‐pure water DNase/RNase‐Free was purchased from Bio‐Lab, Jerusalem.

### Tip Functionalization

The AFM tips were cleaned with the piranha solution for 10 min, followed by rinsing in boiling pure water, and treated by UV‐Ozone (UV/Ozone ProCleanerTM, Bioforce Labs) for 15 min with controlled humidity. The freshly cleaned tips were immediately incubated in a solution of MAL‐PEG‐Silane with a concentration of 1.25 mg mL^−1^ in toluene for 3 hours. After cooling for at least 10 min, the PEG‐modified tips were rinsed by toluene, 2‐propanol, and then Ultra‐pure water. The superparamagnetic iron oxide nanoparticles (5 mg/mL) were mixed with D/L‐cysteine (20 mmol L^−1^) in Ultra‐pure water with a volume‐volume ratio of 3:1. The mixture was subsequently sonicated for 3 hours and then kept in a glove box filled with nitrogen overnight. Then the solution was centrifuged at 17k RCF for 15 min. The centrifuge was repeated two or three times. After centrifuge, 5 mmol L^−1^ of TCEP was added to the nanoparticle‐cysteine complex with a volume‐volume ratio of 1:4. Finally, the PEG‐modified AFM tips were incubated in the cysteine‐modified nanoparticle solution.

### Atomic Force Microscopy Based Force Spectroscopy

The force spectroscopy was conducted in contact mode using a NanoWizard 3 (Bruker‐JPK). The chemically modified AFM tip was slowly approached to the surface at a speed of 1 µm s^−1^ to avoid damaging the attached chiral nanoparticle. In the force measurements, the tip is extended to the surface at a speed of 1 µm s^−1^ until an applied force of 100–200 pN was reached, then held on surface for 100 ms and then retracted at a speed of 0.4 µm s^−1^. The calibration of the optical deflection signal of the AFM tip was performed on the Ni/Ti or Ni/Al_2_O_3_ covered silicon substrate without magnetization. At contact, the sensitivity value of the tip was calibrated from the slope of deflection (V) versus distance (vertical, z motion) curve. After lifting the tip to a separation of 500 µm, thermal tune of the cantilever was used to deduce the spring constant.^[^ ^32^ ^]^ More than 1000 force versus distance curves were recorded. Force curves between one SPION coated with molecules of one (D or L) handedness under mag up and mag down were done using the same tip. Only force curves showing significant pulling events were analyzed by the JPK analysis software (see Figure S17, Supporting Information). The external magnetic field was 0.3 T.

## Conflict of Interest

The authors declare no conflict of interest.

## Author Contributions

R.N. conceived this project. Q.Z. and R.N. designed the experiment. Q.Z. performed the AFM experiment and analyzed the data. S.R.C. guided the AFM measurement and the data analysis. O. B. performed TEM and analyzed the data. J.F. conducted the theoretical calculation. All the authors wrote the manuscript.

## Supporting information

Supporting Information

## Data Availability

The data that support the findings of this study are available from the corresponding author upon reasonable request.
