# Supplementary material for: Magnetic Monopole‐Like Behavior in Superparamagnetic Nanoparticle Coated With Chiral Molecules
Source: Small. 2024 Aug 29;20(48):2406631. doi: 10.1002/smll.202406631 (PMC11600687; doi:10.1002/smll.202406631)
Supplement: Supplementary file 1 — Supporting Information [file SMLL-20-2406631-s001.docx]

Supporting Information

Magnetic Monopole-like Behavior in Superparamagnetic Nanoparticle Coated with Chiral Molecules

Qirong Zhu, Sidney R. Cohen, Olga Brontvein, Jonas Fransson,* and Ron Naaman*

**Table of Contents**

1. Atomic force microscopy measurements
2. X-ray photoelectron spectroscopy measurements
3. Transmission electron microscopy measurements
4. X-ray diffraction measurements
5. Scanning electron microscopy measurements
6. Circular dichroism (CD) measurements
7. Force spectroscopy control experiments
8. Superconducting quantum interference device (SQUID) measurements
9. Contact area between one chiral nanoparticle and the nickel substrate

**1. Atomic force microscopy (AFM) measurements**

AFM was used to characterize the D-cysteine monolayers. The substrate used for adsorption of the chiral molecules was silicon/chromium (10 nm)/gold (120 nm) (Cr/Au). Figures S1a and S1b depict the topography and corresponding phase images. This substrate was incubated in D-cysteine (D-Cys) solution (20 mmol/L in Ultra-pure water) for 24 hours. The thiol group from the D-Cys molecule can chemically bind to the gold surface and thus form a monolayer for which topography and phase images are shown in Figures S1c and S1d. The presence of D-Cys is seen both by the larger grain size in topography and the phase contrast. A solution of D-Cys coated superparamagnetic iron oxide nanoparticles (SPIONs) was drop-cast onto the gold surface and incubated at 100% relative humidity for 24 hours. After rinsing the sample with Ultra-pure water, the sample was sonicated for a few seconds to remove physically absorbed species. Figures S1e and S1f show the topography and phase images of D-Cys SPIONs on the gold surface.

**
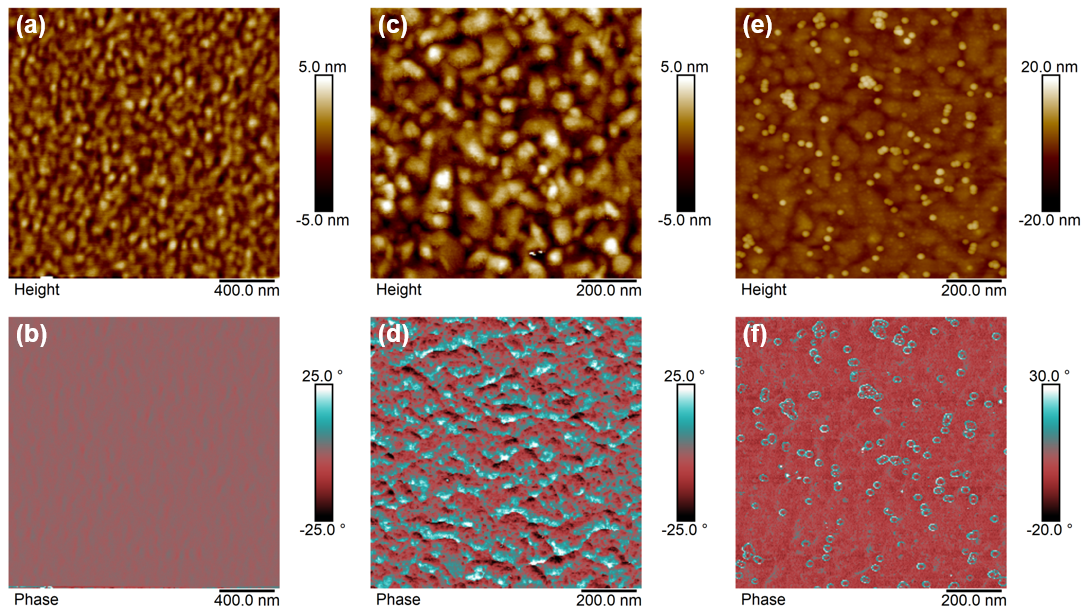
**

**Figure S1.** AFM topography and phase images of a bare gold surface (a and b), D-Cys monolayer on the gold surface (c and d) and D-Cys modified SPIONs (e and f).

The SPIONs were purchased from Sigma-Aldrich (5 mg/mL in H_2_O). The nominal diameter is 10 nm which was verified by AFM and transmission electron microscopy (TEM) measurements. Height profiles from Figure S1e are shown in Figure S2. The heights of ten randomly selected particles give an average value for the chiral SPION nanoparticle diameter of. 10.6±0.4 nm, consistent with the rated value.

**

**

**Figure S2.** Height profiles of ten chiral SPIONs which were randomly chosen from Figure S1e. The mean height and standard error are 10.6±0.4 nm.

The thicknesses of substrate films used were characterized by AFM. The substrate is boron-doped p-type silicon. Ti and Ni layers are evaporated onto the substrates using an Odem High throughput KW E.B.Gun system designed for electron beam and thermal deposition (Scientific Applications Ltd.). A Fiji F200 Plasma atomic layer depostion (ALD) system was used to prepare the Al_2_O_3_ layer. For all substrates, a 10 nm Ti adhesion later was evaporated at a rate of 0.5 Å/s. The 120 nm Ni magnetic layer was evaporated on this layer at a rate of 0.5 Å/s for 30 nm, then a rate of 1.0 Å/s for another 30 nm and finally at 1.5 Å/s for the remaining 60 nm. The non-magnetic Ti layers were evaporated on the Ni at a rate of 0.5 Å/s. The nominal 8 nm Al_2_O_3_ deposition on the Ni surface was produced using trimethylaluminium (TMA) and water precursors at a temperature of 180°C and with 80 ALD cycles. The nominal Ti thicknesses of 2 nm and 5 nm (Ti2 and Ti5) and Al_2_O_3_ (8 nm) are values derived from the calibration of the deposition systems, however the actual heights were verified by AFM (Figures S3, S4 and S5). Ten profiles were randomly chosen from each interface from which mean heights with standard errors were calculated. The thicknesses of Ti2, Al_2_O_3_ and Ti5 were 2.9±0.1 nm, 8.9±0.2 nm and 9.4±0.2 nm, respectively.

**
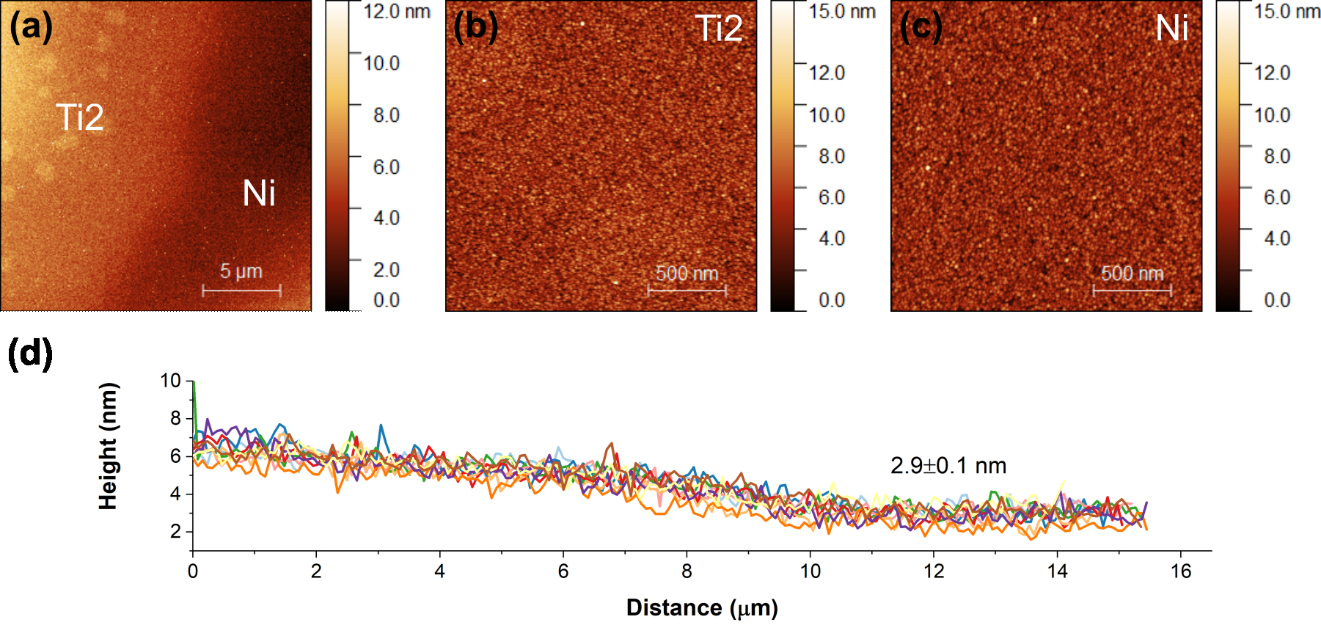
**

**Figure S3.** AFM topographies of (a) Ni/Ti2 interface, (b) Ti surface and (c) Ni surface. (d) Ten profiles were randomly collected from (a). The mean thickness of Ti2 with the standard error is 2.9±0.1 nm.

**
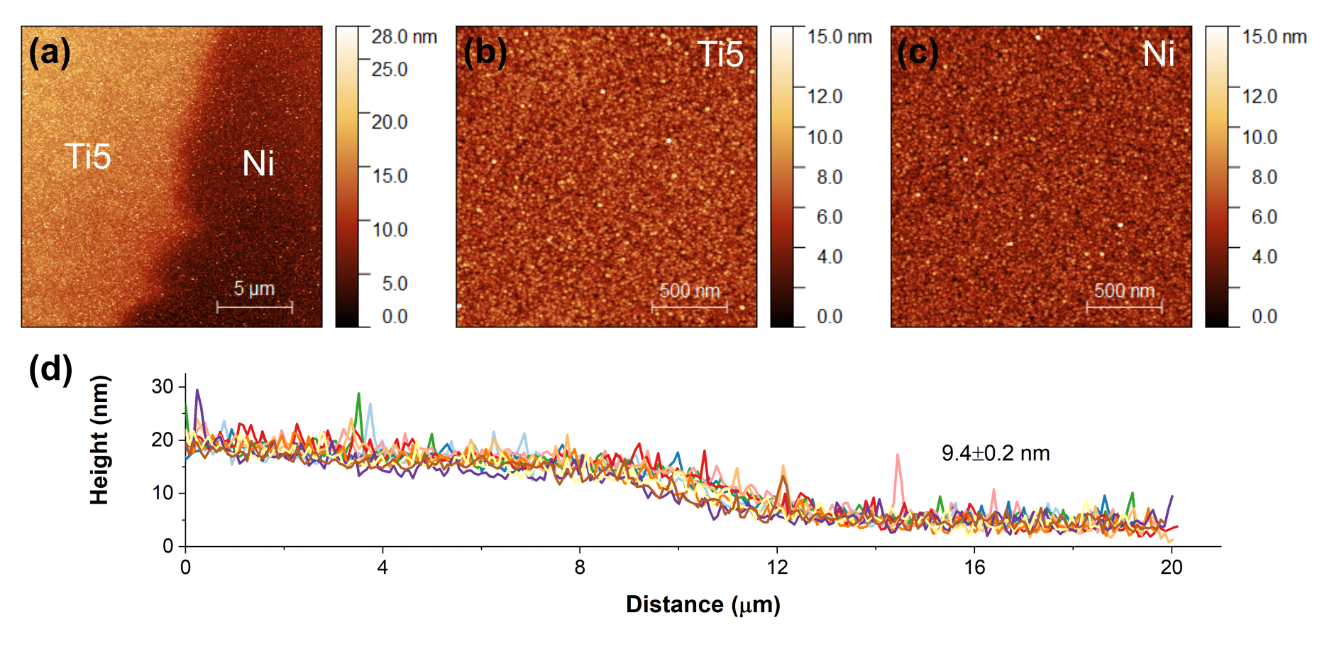
**

**Figure S4.** AFM topographies of (a) Ni/Ti5, (b) Ti5 surface and (c) Ni surface. (d) Ten profiles were randomly chosen from (a). The mean thickness of Ti5 with the standard error is 9.4±0.2 nm.

**
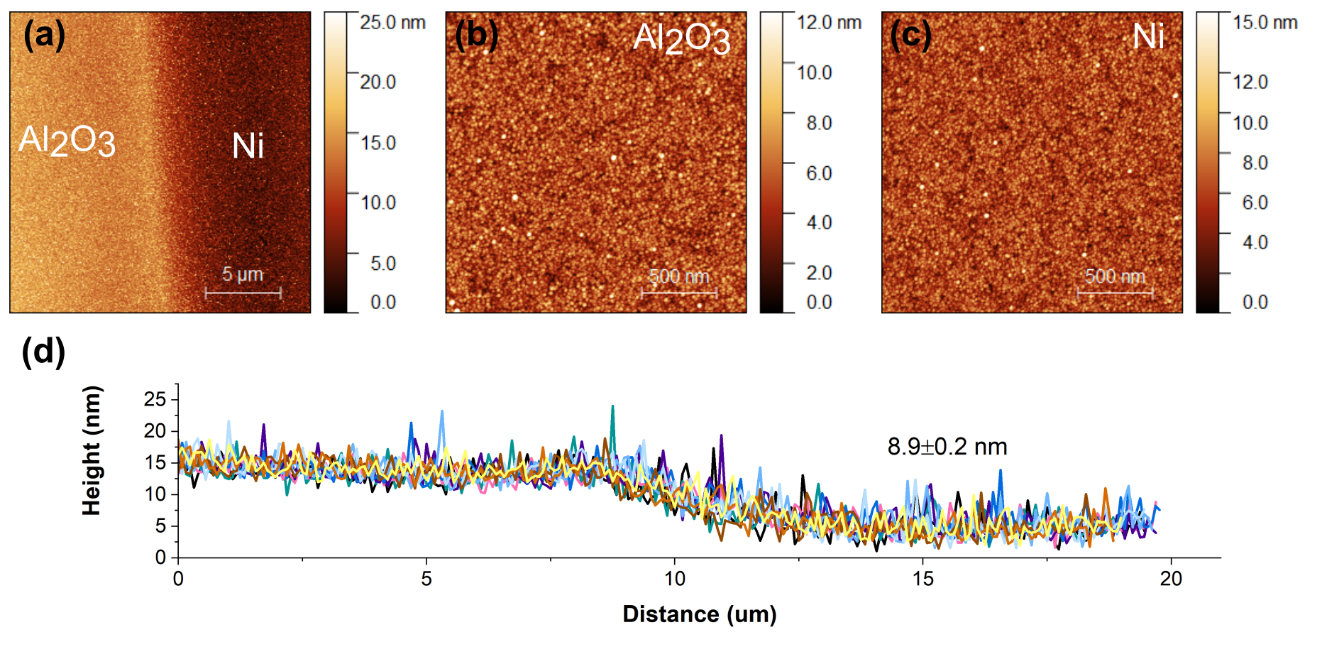
**

**Figure S5.** AFM topographies of (a) Ni/Al_2_O_3_, (b) Al_2_O_3_ surface and (c) Ni surface. (d) Ten profiles were randomly chosen from (a). The mean thickness of Al_2_O_3_ with the standard error is 8.9±0.2 nm.

**2. X-ray photoelectron spectroscopy (XPS) measurements**

Surface elemental and oxidation state composition were measured using Axis Supra XPS (Kratos) with monochromatic Al α (1486.7 eV) x-ray line, with photoelectrons detected at take-off angle of 90°. Metallic Ti and its oxides (Ti^+^, Ti^2+^, Ti^3+^ and Ti^4+^) were observed in both Ti films (Figures S6a and S6b)^[1]^ The ALD-grown Al_2_O_3_ film characterized by XPS (shown in Figure S6c) presented the property of insulating based on Al-O bond Al-OH in Al 2p.^[2]^ The Fe_3_O_4_ nanoparticles before and after chemical modification (SPION and L-Cys SPION) were characterized by XPS after being drop-casted onto gold coated silicon substrates(Figures S7 and S8). The existence of O-C-O in the C 1s region, -NH_3_^+^ in the N 1s region, unbound -SH in S 2p as well as C-O and Fe-O in the O 1s region proves the chemical attachment of L-cysteine molecules onto Fe_3_O_4_ nanoparticles (SPIONs).^[3-5]^ Fe 2p of Fe_3_O_4_ nanoparticles before and after modification present Fe^2+^ species, Fe^3+^ species and two satellites peaks.^[6]^

**
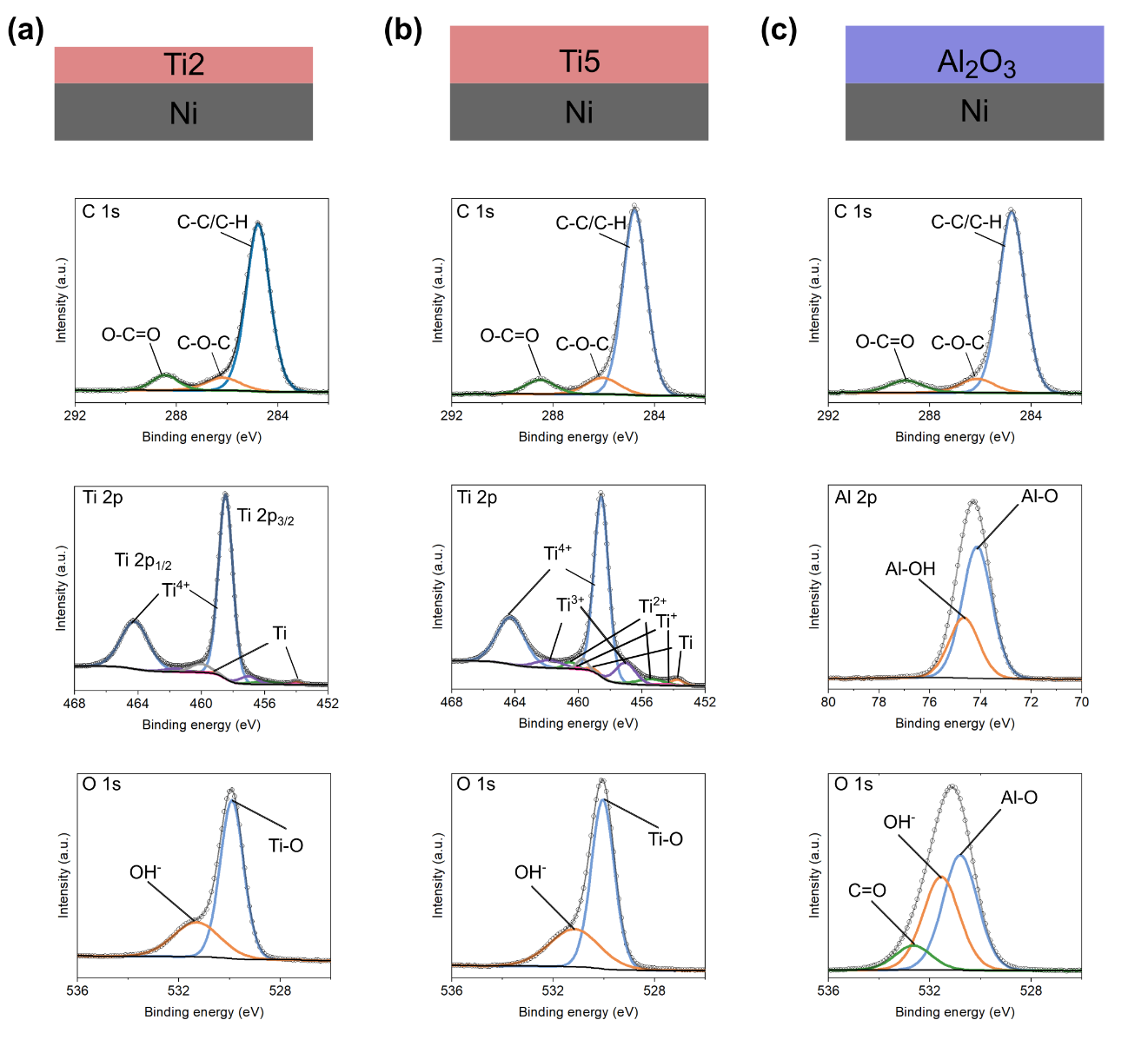
**

**Figure S6.** XPS spectra of the three substrates. Ni/Ti2 (a) and Ni/Ti5 (b) presents C 1s, Ti 2p and O 1s spectra. (c) for Ni/Al_2_O_3_ presents C 1s, Al 2p and O 1s spectra.

**
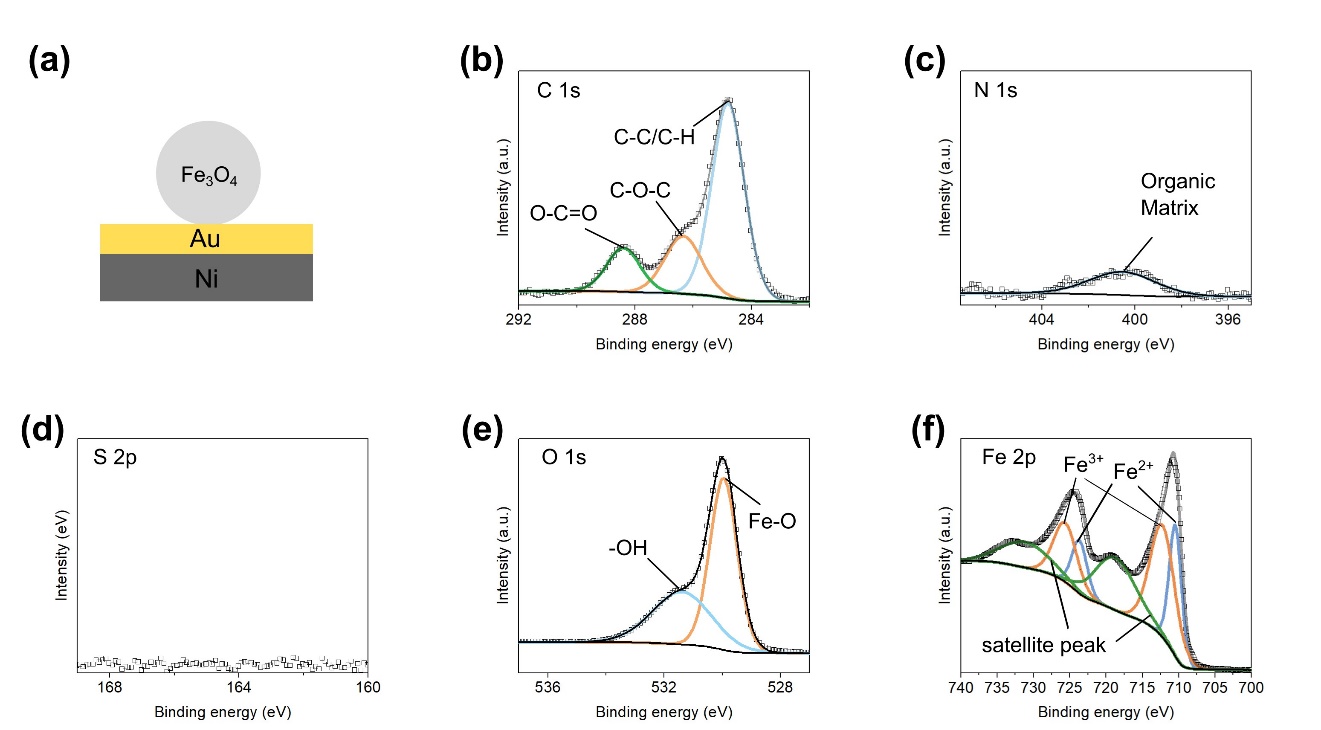
**

**Figure S7.** XPS spectra of unmodified SPIONs on a gold coated silicon substrate: (a) schematic diagram, (b) C 1s, (c) N 1s, (d) S 2p, (e) O 1s and (f) Fe 2p.

**
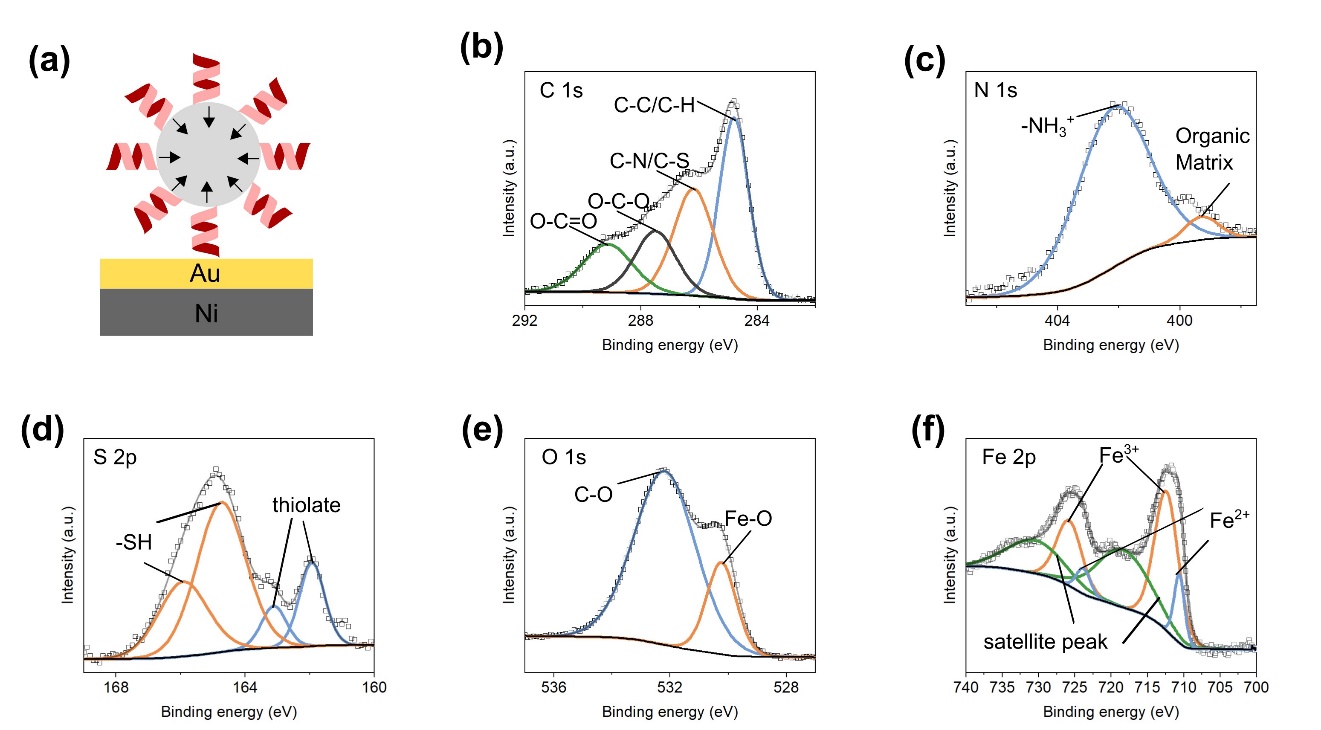
**

**Figure S8.** XPS spectra of L-Cys SPIONs on a gold coated silicon substrate: (a) schematic diagram, (b) C1s, (c) N 1s, (d) S 2p, (e) O 1s and (f) Fe 2p.

**3. Transmission electron microscopy (TEM) measurements**

Talos F200X G2 (Thermo Fisher Scientific) was used for characterization of SPIONs before and after modification. The main objective here is to first analyze the mean diameter by high resolution TEM and then perform elemental mapping by energy dispersive spectroscopy (EDS). From figures S9 and S11, the mean diameters of SPION and L-Cys SPION are 8.6 +/- 0.2 nm and 10.8 +/- 0.2 nm, respectively. Comparing Figures S10 with S12, the presence of sulfur (S) after L-cysteine modification proves the successful attachment to SPION. This EDS data is consistent with the XPS results. The TEM -selected area electron diffraction (SAED) measurement shown in Figure S13 polycrystalline ED pattern of cubic Fe_3_O_4_.^[7]^

**
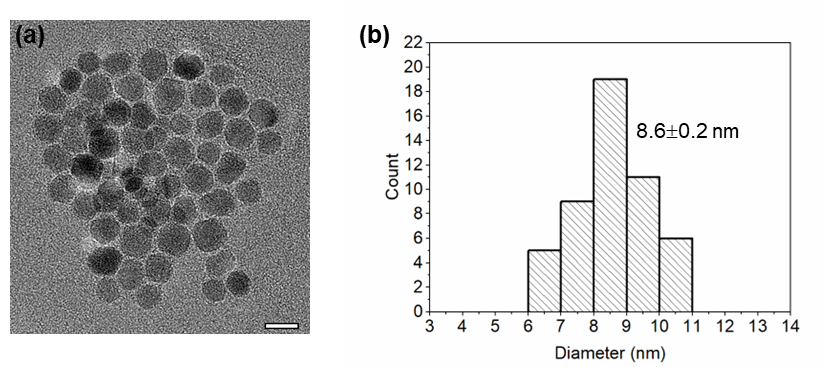
**

**Figure S9.** TEM image (a) and size histogram (b) of SPIONs before chemical modification. Scale bar in (a) is 10 nm.

**
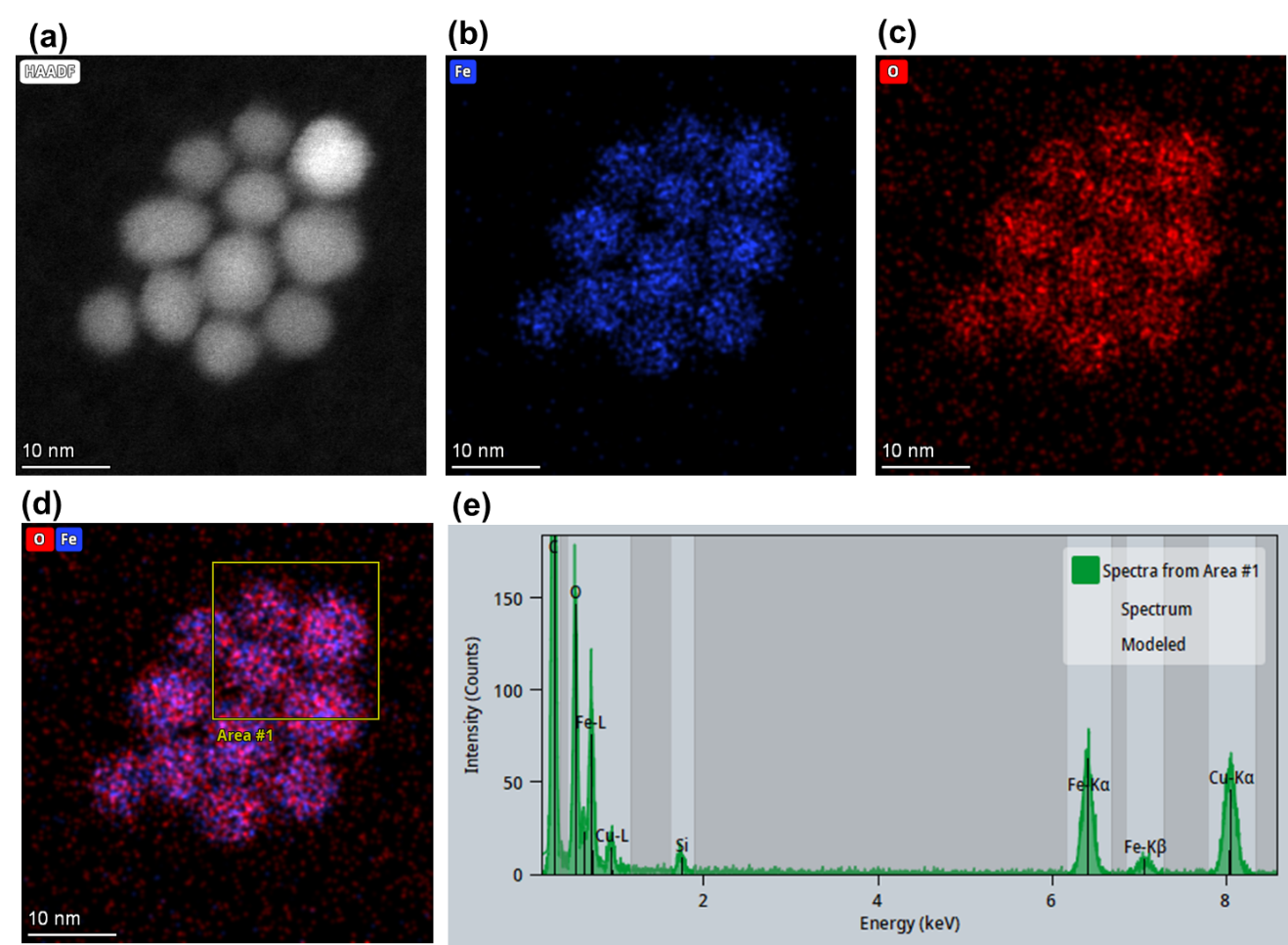
**

**Figure S10.** The HAADF STEM image (a), the EDS maps of corresponded elements (b-c) of SPIONs before chemical modification. The overlap of presented elements (d) with EDS spectrum (e) corresponded to the marked area in (d).

**
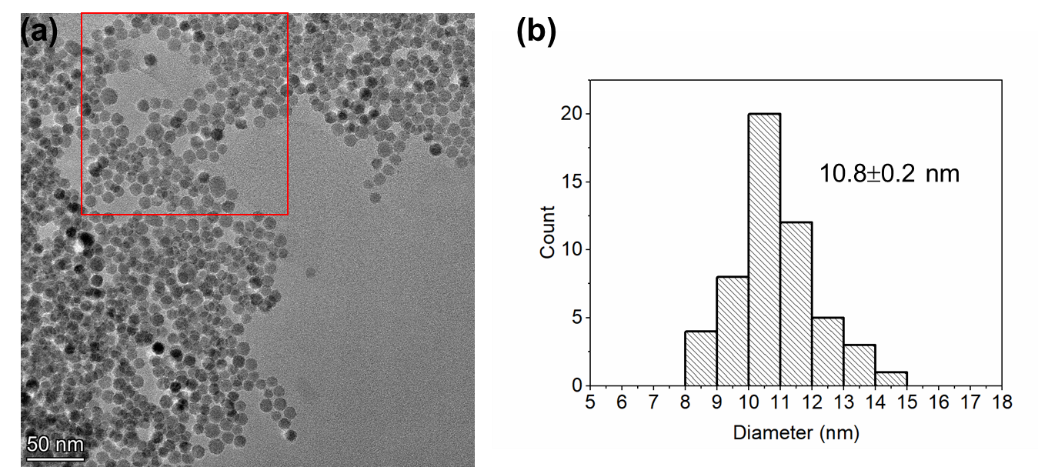
**

**Figure S11.** TEM image (a) and corresponding size analysis (b) of L-Cys SPIONs. The histogram in b was extracted from the red rectangle in a.

**
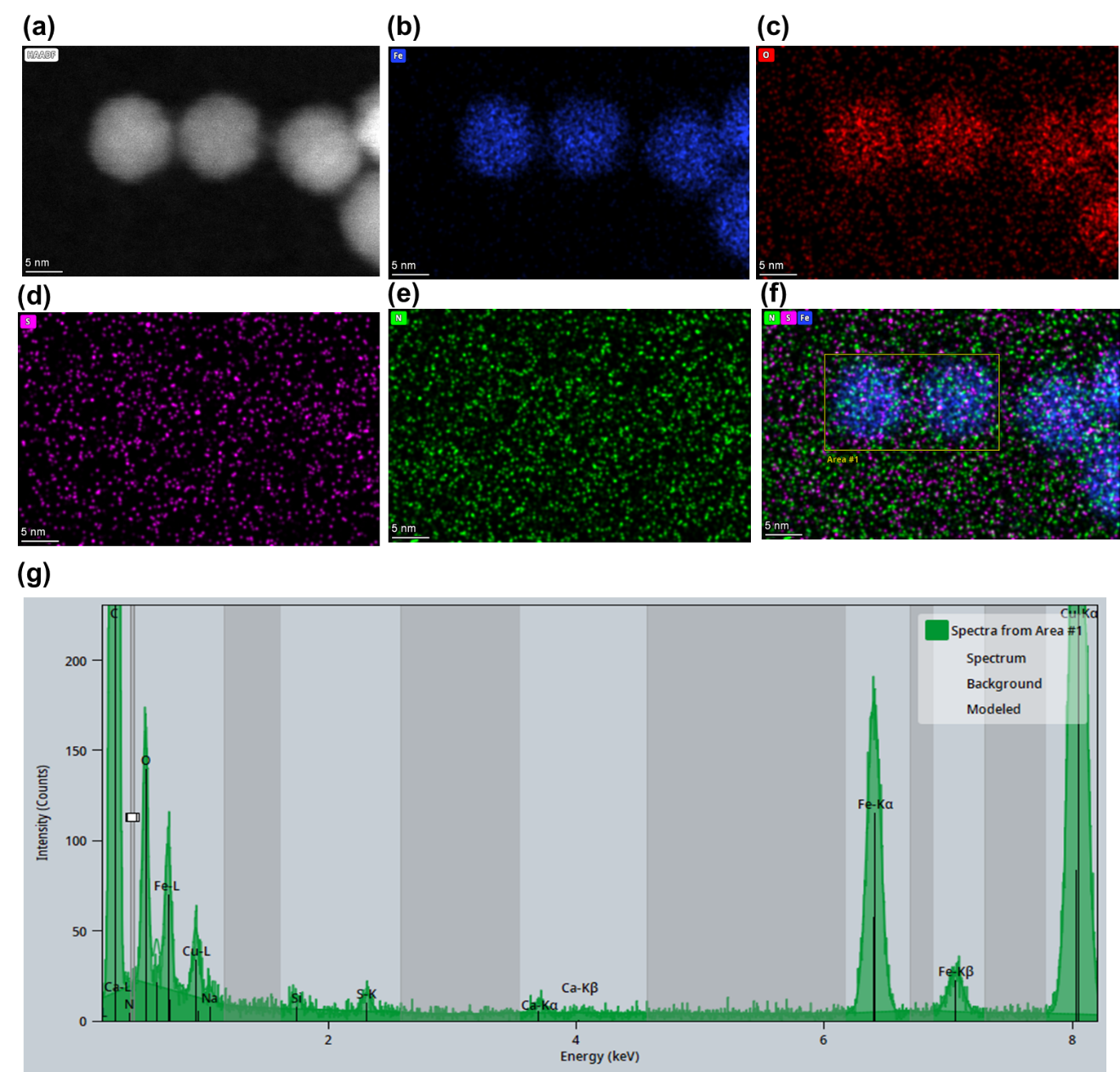
**

**Figure S12.** The HAADF STEM (a) image and the EDS elemental maps of elements (b-e) of L-Cys SPIONs. The overlap of Fe, N and S (f) with EDS spectrum (g) corresponded to the marked area in (f).

**
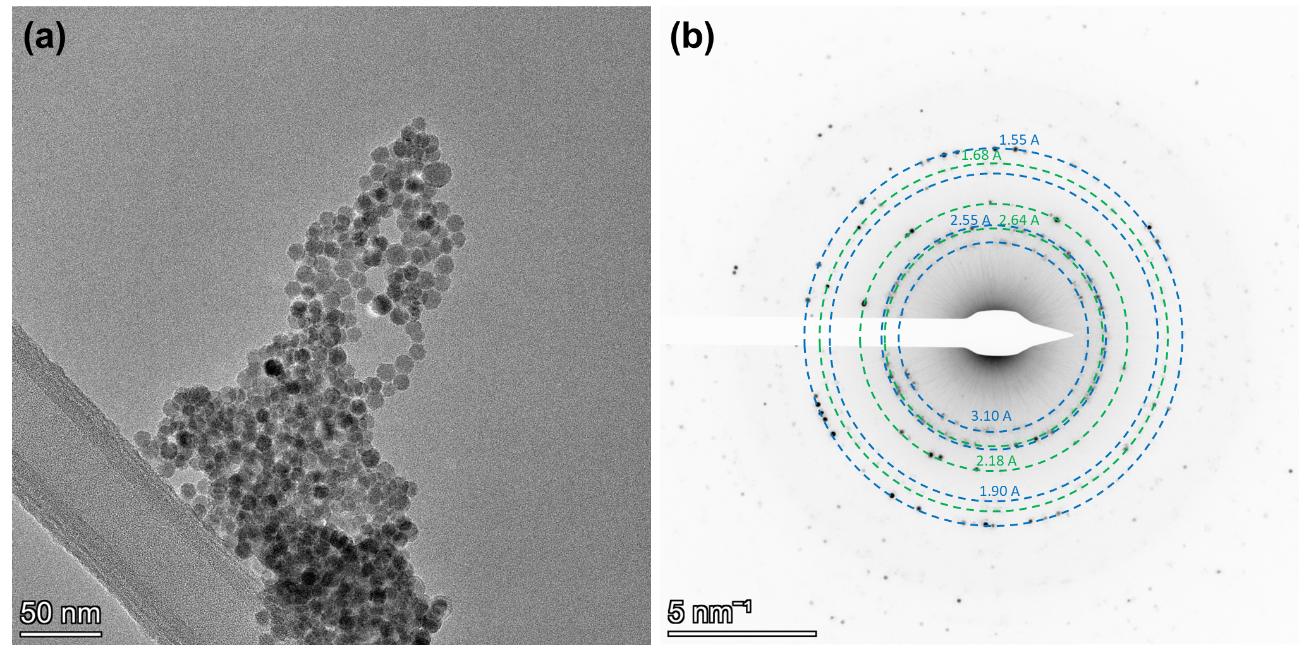
**

**Figure S13.**  The TEM image (a) and SAED measurements (b) of L-Cys SPIONs.

**4. X-ray diffraction measurements**

X-Ray Powder Diffractometer D8 Advance (Bruker AXS) was used for characterizing the structural information of SPIONs and L-Cys SPIONs. Figure S14a shows the diffraction pleaks which can be indexed to the (220), (311), (400), (440) and (533) planes of cubic Fe_3_O_4_ (JCPDS# 65-3107).^[8]^

**
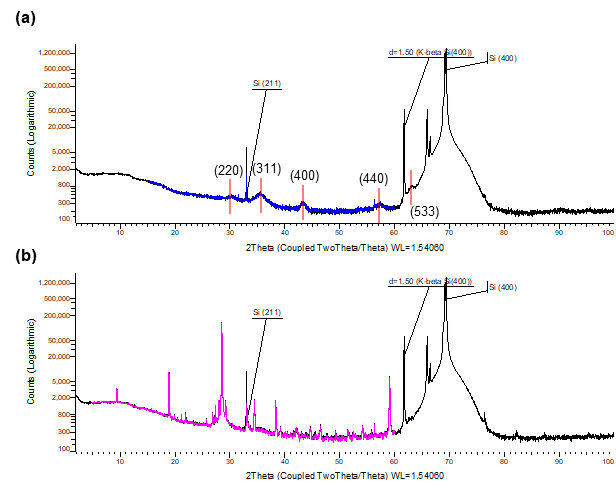
**

**Figure S14.** XRD pattern of SPIONs (a) and L-Cys SPIONs (b) on silicon substrates. L-Cys SPIONs were functionalized with a concentration of 200 mmol/L L-cysteine.

**5. Scanning electron microscopy (SEM) measurements**

A high resolution SEM (Gemini SEM 500) was used for characterization of L-Cys SPIONs attached to an AFM tip. Whereas the polyethylene glycol (PEG) alone cannot be unequivocally detected in the SEM, the PEG-linked chiral SPIONs can be directly discerned by backscattered electrons in the SEM. Figures S15a and S15b display the SEM and its corresponding backscattered electron images. The bright spots in Figure S15b are L-Cys SPIONs which were chemically attached to the AFM tip through the PEG linkers.

**
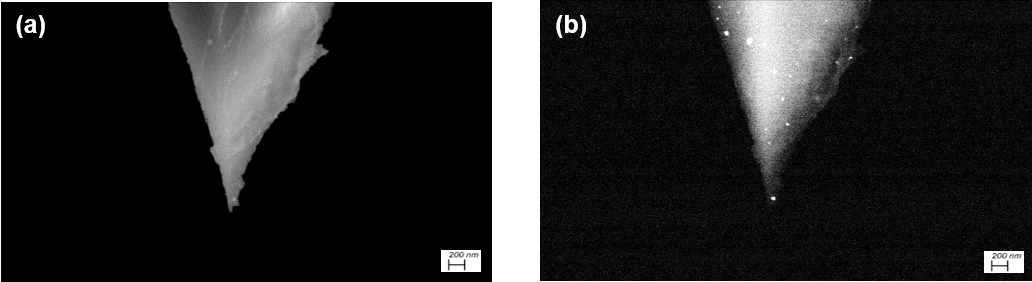
**

**Figure S15**. SEM (a) and corresponding backscattered electrons’ (b) images of L-Cys SPIONs attached to a sharp AFM tip through the polyethylene glycol (PEG) linkers.

**6. Circular dichroism (CD) measurements**

The D-Cys and L-Cys were both dissolved in pure water at a concentration of 20 mmol/L. Then the solution was diluted to 5 mmol/L. The D-Cys and L-Cys were measured by CD. The CD spectra of both D-Cys and L-Cys show a peak at 201 nm (shown in Figure S16a). Figure S16b is the corresponding absorbance of D-Cys and L-Cys. After D/L-Cys were attached to the SPIONs, two new peaks at 211 nm and 255 nm appear due to the formation of a new Cys-SPION complex (see Figure S16c). The absorbance of Cys-SPION is shown in Figure S16d. In the case of Cys-SPION, the D/L-Cys solution was prepared with a concentration of 200 mmol/L because the peak is not detectable at the lower concentrations.

**
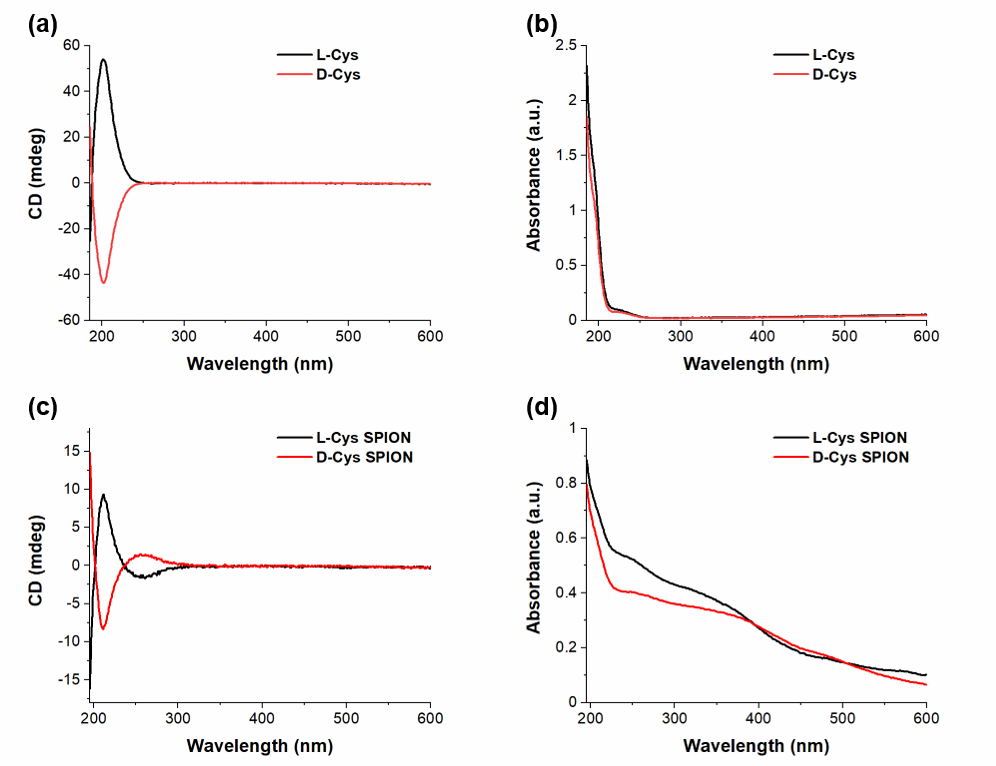
**

**Figure S16**. CD spectra (a and c) and corresponding absorbance (b and d) of D/L-Cys and D/L-Cys SPION.

**7. Force spectroscopy control experiments**

AFM can be used to provide confirmation of successful attachment on SPION and PEG linker independent of the SEM studies described above. Figure S17 is an example of how the experiment is performed in AFM-based force spectroscopy. Figure S18 presents a random selection of 100 raw force curves between the L-Cys SPION and the Ni/Ti (120/2.9 nm) substrate. To provide more insight on the results presented in Figures 2 and 3, Figures S19 and S20 show the pulling force versus distance (height) distribution.


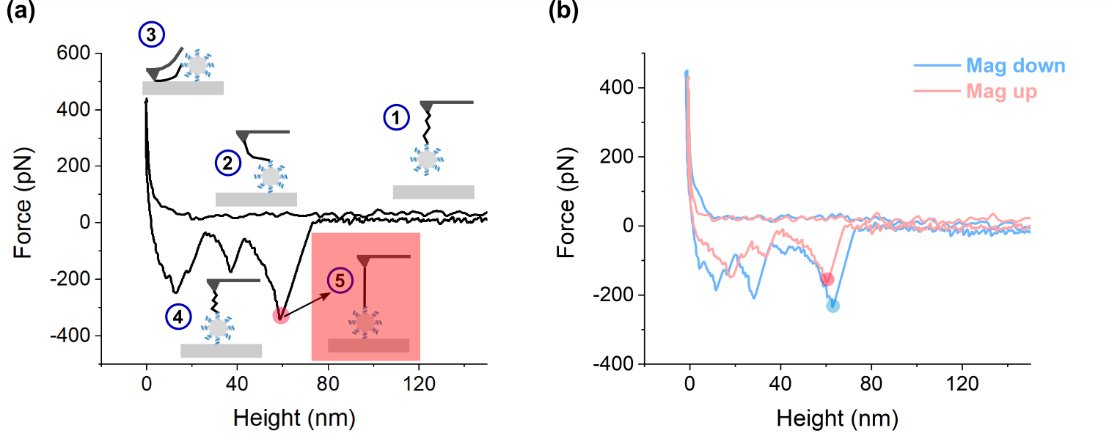


**Figure S17**. (a) A representative force vs. distance curve of a chiral SPION attached to an AFM tip interacting with the ferromagnetic substrate. Five stages are depicted: (1) the AFM tip with a chiral SPION approaching the substrate, (2) the chiral SPION in contact with the substrate, (3) the AFM tip and the chiral SPION in repulsive contact with the substrate causing the AFM cantilever to bend, (4) the AFM tip retracting from the substrate while the chiral SPION remains in contact with the substrate and (5) the breaking point (red spot) where the chiral SPION separates from the substrate. The pulling force reported is the force at the breaking point). More than 163 curves for each magnet direction which show significant pulling events are analyzed for each case. (b) Representative force versus distance curves under mag up and mag down. The pulling events were analyzed for the distance range of 30 nm to 120 nm from the surface (height 0).


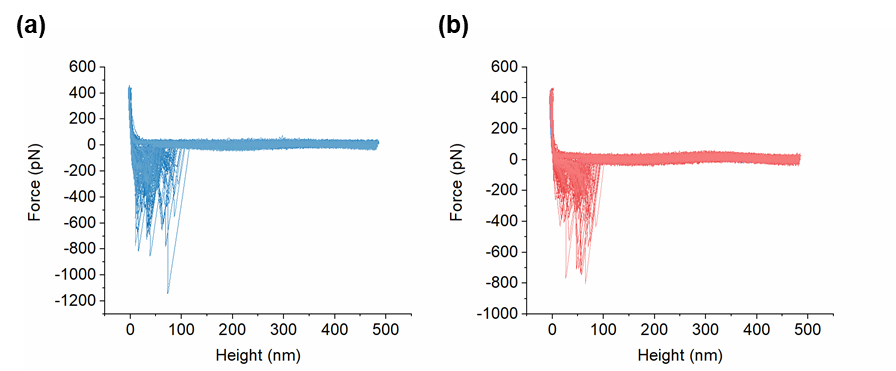


**Figure S18**. A random selection of 100 raw force curves for the L-Cys SPION interacting with the Ni/Ti (120/2.9 nm) substrate under mag down (a) and mag up (b) conditions.


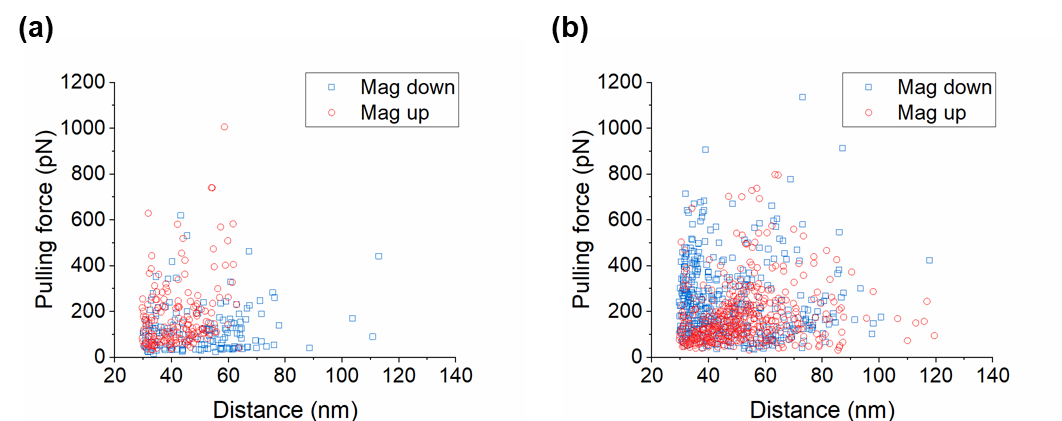


**Figure S19**. Pulling force (pN) versus distance (nm) for the data of Figure 2: (a) D-Cys SPION and (b) L-Cys SPION interacting with the Ni/Ti (120/2.9 nm) substrate.


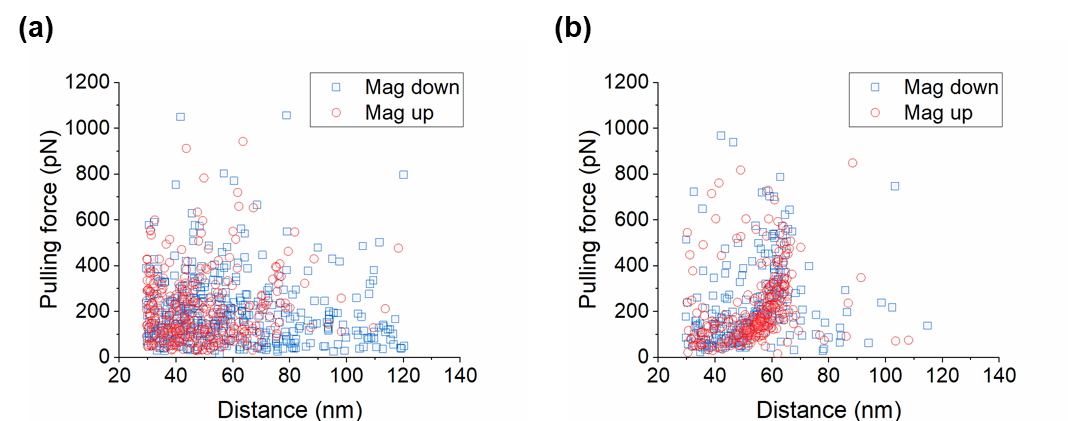


**Figure S20**. Pulling force (pN) versus distance (nm) of Figure 3: (a) D-Cys SPION and (b) L-Cys SPION interacting with the Ni/Al_2_O_3_ (120/8.9 nm).

The main text reports the representative pulling force for the Ni/Al_2_O_3_ substrate (8.9±0.2 nm Al_2_O_3_) and the Ni/Ti substrate (2.9±0.1 nm Ti). The averaged difference between up/down magnet orientation force values and their uncertainties are shown in Figure S21.





**Figure S21**. The average force difference between up/down magnet orientation of the Ni/Al_2_O_3_ substrate (8.9 nm Al_2_O_3_) and the Ni/Ti substrate (2.9 nm Ti): 15.4±10.0 pN (collected from 5 samples) and 51.6±7.9 pN (collected from 4 samples), respectively. The force difference and its standard error are both average values of the force between the D/L-Cys SPION and the corresponding substrate.

The force interaction between the PEG linker attached to an AFM tip and a Si/SiO_2_/Ti(10 nm)/Ni(120 nm)/Al_2_O_3_(8.9±0.2 nm) substrate (Ni/Al_2_O_3_ substrate) was measured under both magnet directions as a control. Referring to previous force spectroscopy studies of PEG linkers, we considered pulling forces ranging from 0 to 150 pN and extension length (stretching distance) between 25 nm to 65 nm.^[9,10]^

**
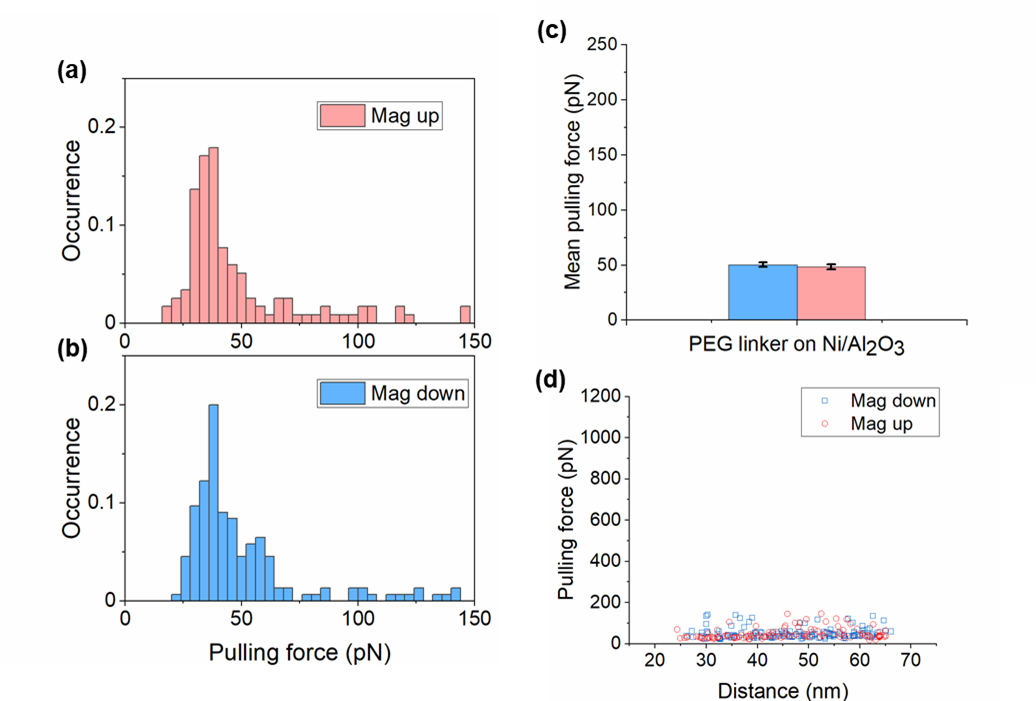
**

**Figure S22**. AFM-based force spectroscopy measurements of the PEG linker on the Ni/Al_2_O_3_ substrate.

(a) Histogram of the pulling force between the PEG linker and the Ni/Al_2_O_3_ substrate under up magnetization (mag up, red color).

(b) Histogram of the pulling force between the PEG linker and the Ni/Al_2_O_3_ substrate under down magnetization (mag down, blue color).

The pulling force was recorded at a retracting speed of 400 nm s^−1^. Error bars represent the standard error. The Al_2_O_3_ film is insulating which partially blocks the charge and spin injection from the substrate.

(c) Mean pulling force between the PEG linker and the Ni/Al_2_O_3_ substrate.

(d) Pulling force (pN) versus distance (nm) plot between the PEG linker and the Ni/Al_2_O_3_ substrate for all data acquired.

Figure S22c shows that the mean pulling force (MPF) values of the PEG linker for mag up and mag down are 48.4±2.4 pN and 50.3 ± 2.0 pN, respectively. Corresponding histograms and distribution of pulling force versus distance are displayed Figures S22a, S22b and S22d. Figure S23 presents the MPF values of the SPION coated with 3-mercaptopropionic acid (3-MPA) molecules and interacting with a Ni/Ti (120/2.9 nm) substrate. for mag up and mag down are 223.6 ± 17.9 pN and 224.1 ± 7.4 pN, respectively. Corresponding histograms and distribution of pulling force versus distance are shown in Figures S23a, S23b and S23d.


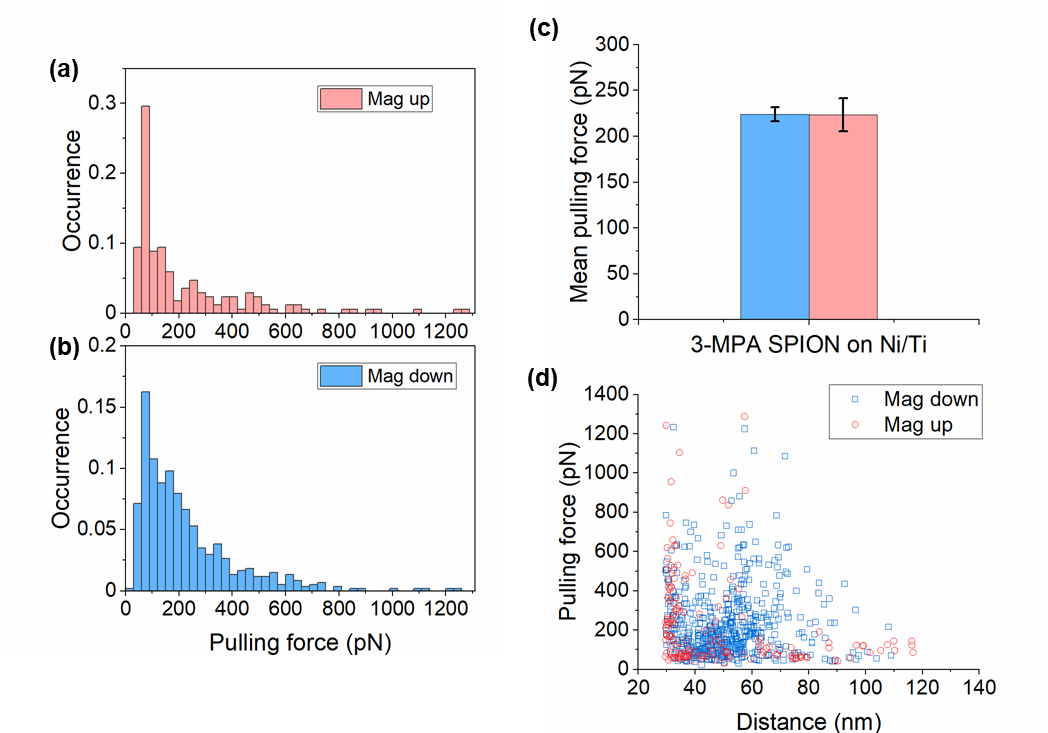


**Figure** **S23**. AFM-based force spectroscopy of 3-MPA coated SPION interacting with the Ni/Ti (120/2.9 nm) substrate. (a) Histogram of the pulling forces under up magnetization.

(b) Histogram of the pulling force under down magnetization. The pulling force was recorded at a retracting speed of 400 nm s^-1^. Error bars represent the standard error.

(c) Bar chart of mean pulling force and standard error (red-mag up, blue-mag down).

(d) Pulling force (pN) versus distance (nm).

As an additional control, the thickness of titanium (Ti) between the Ni layer and the chiral SPION was increased from 2.9±0.1 nm (Figure 2 in the main text) to 9.4±0.2 nm. Figure S24 presents the force interaction between the Si/SiO_2_/Ti(10 nm)/Ni(120 nm)/Ti(9.4nm) and the chiral SPION. The MPF values for L-Cys SPION under mag up and mag down are 169.9±10.4 pN and 171.9±9.6 pN, respectively. Corresponding histograms and distribution of pulling force versus distance are shown in Figures S24a, S24b and S24d.


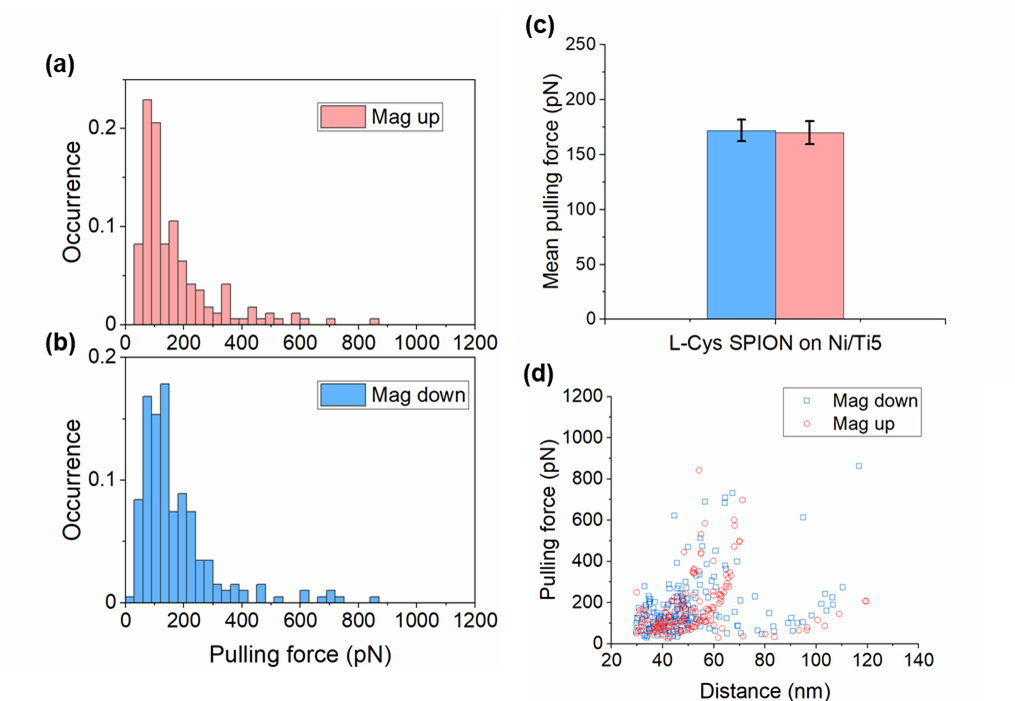


**Figure S24**. AFM-based force spectroscopy measurements of L-Cys SPION interacting on Ni (120 nm) /Ti (9.4±0.2 nm) substrates (Ni/Ti5) in PBS buffer.

(a) Histogram of the pulling force between the PEG linker and the Ni/Ti5 substrate under up magnetization (mag up, red color).

(b) Histogram of the pulling force between the PEG linker and the Ni/Ti5 substrate under down magnetization (mag down, blue color).

The pulling force was recorded at a retracting speed of 400 nm s^−1^. Error bars represent the standard error.

(c) Mean pulling force between L-Cys SPION and the Ni/Ti5 substrate.

(d) Pulling force (pN) versus distance (nm) distribution between the PEG linker and the Ni/Ti5 substrate.

The AFM-calibrated thickness of Ti5 is 9.4±0.2 nm.

**8.** **The superconducting quantum interference device (SQUID) measurements**

Our SPIONs were chemically modified by D-Cys and L-Cys. In our work, we mainly focus on the nanoscale force interaction between a single chiral SPION and the ferromagnetic substrate. Here, we demonstrate magnetic moment measurements of randomly packed chiral SPIONs in Figure S25. It shows zero coercivity and no remnant field in our data. Previous work has shown that chiral molecules embedded SPION at two sides or covered symmetrically over the outer surface has no significant magnetization because the symmetry plane is not broken and no net stable magnetization is obtained.^[11]^ Our observation is consistent with that work.

**
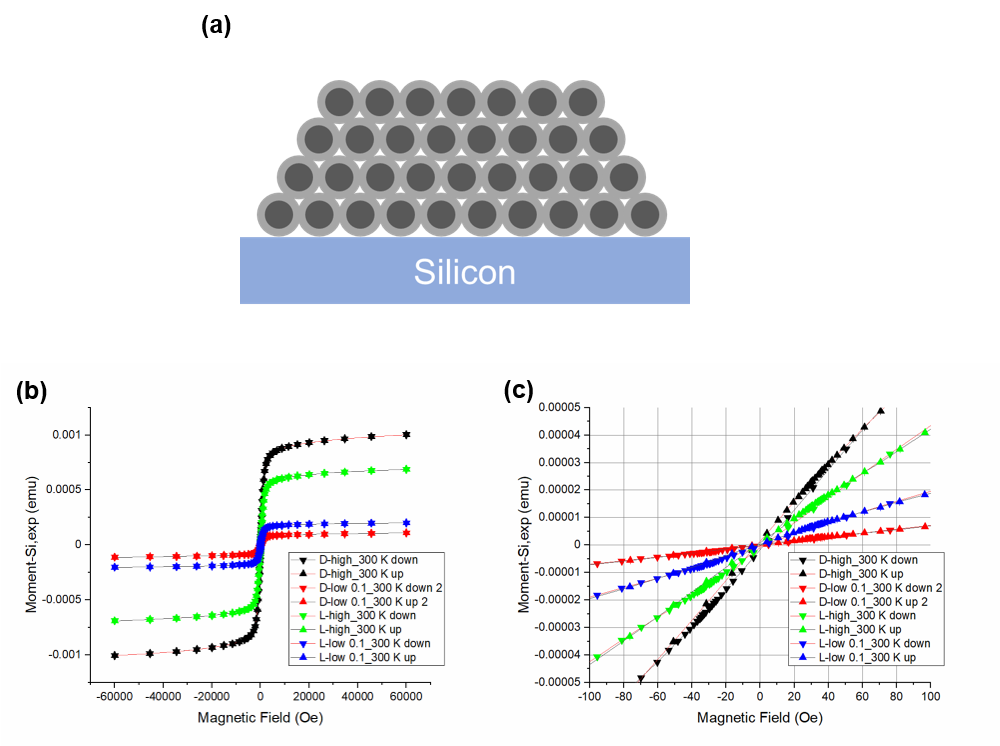
**

**Figure S25**. SQUID measurements of randomly packed chiral SPIONs.

(a) Schematic diagram of the sample for the SQUID measurements: D/L-Cys modified SPIONs densely packed on the silicon chip.

(b) SQUID measurements of the magnetization hysteresis loop for D-Cys modified SPIONs (low or high density) and LCys modified SPIONs (low or high density) with magnetic field ranging from -60000 Oe to +60000 Oe.

(c) The zoomed-in SQUID data with magnetic field ranging from -100 Oe to +100 Oe.

**9. Contact area between one chiral nanoparticle and the nickel substrate**

There are several contact mechanics models, such as Hertz Theory^[12]^, Johnson-Kendall-Roberts (JKR) model^[13]^ and Derjaguin-Muller-Toporov (DMT) model^[14]^ to determine surface forces, contact area and deformation. The Hertz model neglects the adhesion force. Both the JKR model and DMT models consist of a deformation contribution based on the Hertz model and an adhesion component owing to the surface energy. The JKR model is more suitable for large spheres with compliant samples and large adhesion. The DMT model applies for relatively small spheres with stiff surfaces with low adhesion. The Maugis elasticity parameter provides the measure for which regime applies.^[15]^ For our experimental parameters, the Maugis elasticity parameter is calculated to be 0.1, therefore the behavior should follow the DMT model.

In our experiment, the SPION is chemically modified by D/L-Cys. The Young’s modulus of the iron oxide nanoparticles (Fe_3_O_4_) is ~120 GPa.^[16]^ After chemical modification, the organic molecule coated iron oxide nanoparticle has an effective modulus of 2 GPa.^[17]^ The Young’s modulus of nickel is 200 GPa which comprises a rigid surface.^[18,19]^ Therefore, we can model our interaction as an elastic sphere with radius R in contact with a rigid, planar nickel surface (shown in Figure S26).^[20]^


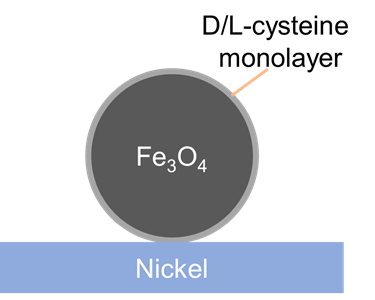


**Figure S26**. Schematic diagram of the D/L-Cys modified iron oxide nanoparticle on a nickel coated surface: a sphere on a flat.

The reduced Young’s modulus E* for the sphere-plane system is^[21]^

$\frac{1}{E^{*}}=\frac{3}{4}(\frac{1-{{}_{s}}^{2}}{E_{s}}+\frac{1-{{}_{f}}^{2}}{E_{f}})$ (1)

where E_s_, E_f_ and ν_s_, ν_f_ are the Young’s modulus and Poisson’s ratio of the sphere and the surface, respectively. The Poisson’s ratio of the nickel is 0.31. The Poisson’s ratio of cysteine coated iron oxide nanoparticle is estimated from a similar organic monolayer on gold surface, which is 0.44.^[22]^ The reduced Young’s modulus calculated from Eq. 1 is 3.3 GPa.

For DMT model,^[23]^ the adhesion force is

$F_{ad}=2\pi R_{p}w_{a}$ (2)

Where R_p_ is the radius of the sphere and w_a_ is the work of adhesion per unit area.

The contact area is found through the formula^[24]^

$\frac{{a_{c}}^{2}}{R_{p}}=\frac{{(F+2\pi R_{p}w_{a})}^{\frac{2}{3}}}{\sqrt[3]{R_{p}{E^{*}}^{2}}}$ (3)

Where a_c_ is the contact radius and F is the loading force.

Thus, the contact area S_c_ is

$S_{c}=\pi{a_{c}}^{2}=\pi{[\frac{R_{p}(F+F_{ad})}{E^{*}}]}^{\frac{2}{3}}$ (4)

The average radius of the iron oxide nanoparticle is 5 nm. The length of D/L-cysteine is estimated to be 0.3 nm.^[25]^ Therefore, the radius of the cysteine modified nanoparticle (R_p_) is 5.3 nm.

During the force measurement, the setpoint for the applied force (F) ranged from 100 pN to 200 pN. The adhesion force is around 200 pN. Thus, the contact area is from 1.9 to 2.3 nm^2^. The corresponding deformed depth is about 0.06 ~ 0.07 nm. Assuming the coverage of the cysteine monolayer is 100%, there are between 21 to 26 cysteine molecules in contact between the sphere and the surface.

References

[1] K. Ronoh, S. H. Fawaeer, V. Holcman, A. Knápek, D. Sobola, *Vacuum* **2023**, *215*, 112345.

[2] I. Iatsunskyi, M. Kempiński, M. Jancelewicz, K. Załęski, S. Jurga, V. Smyntyna, *Vacuum* **2015**, *113*, 52.

[3] G. Dodero, L. De Michieli, O. Cavalleri, R. Rolandi, L. Oliveri, A. Daccà, R. Parodi, *Colloids Surf. Physicochem. Eng. Asp.* **2000**, *175*, 121.

[4] O. Cavalleri, G. Gonella, S. Terreni, M. Vignolo, P. Pelori, L. Floreano, A. Morgante, M. Canepa, R. Rolandi, *J. Phys. Condens. Matter* **2004**, *16*, S2477.

[5] R. Zhang, T. Leiviskä, *J. Chem. Eng.* **2020**, *385*, 123967.

[6] Q. Ai, Z. Yuan, R. Huang, C. Yang, G. Jiang, J. Xiong, Z. Huang, S. Yuan, *J. Mater. Sci.* **2019**, *54*, 4212.

[7] M. E. Fleet, *Acta Crystallogr., Sect. B: Struct. Sci.* **1981**, *37*, 917.

[8] W. Zhang, K. Banerjee-Ghosh, F. Tassinari, R. Naaman, *ACS Energy Lett.* **2018**, *3*, 2308– 2313.

[9] C. Ray, J. R. Brown, B. B. Akhremitchev, *J. Phys. Chem. B* **2007**, *111*, 1963.

[10] G. Stan, F. W. DelRio, R. I. MacCuspie, R. F. Cook, *J. Phys. Chem. B* **2012**, *116*, 3138.

[11] G. Koplovitz, G. Leitus, S. Ghosh, B. P. Bloom, S. Yochelis, D. Rotem, F. Vischio, M. Striccoli, E. Fanizza, R. Naaman, D. H. Waldeck, D. Porath, Y. Paltiel, *Small* **2019**, *15*, 1804557.

[12] Q. J. Wang, D. Zhu, in *Encycl. Tribol.* (Eds.: Q. J. Wang, Y.-W. Chung), Springer US, Boston, MA, **2013**, pp. 1654–1662.

[13] K. L. Johnson, K. Kendall, A. D. Roberts, *Proc. R. Soc. Lond. A* **1971**, *324*, 301-313.

[14] V. M. Muller, B. V. Derjaguin, Yu. P. Toporov, *Colloids Surf.* **1983**, *7*, 251.

[15] I. Rosenhek-Goldian, S. R. Cohen, *J. Vac. Sci. Technol. A* **2023**, *41*, 062801.

[16] A. Gholizadeh, *J. Am. Ceram. Soc.* **2017**, *100*, 3577.

[17] P. Georgopanos, G. A. Schneider, A. Dreyer, U. A. Handge, V. Filiz, A. Feld, E. D. Yilmaz, T. Krekeler, M. Ritter, H. Weller, V. Abetz, *Sci. Rep.* **2017**, *7*, 7314.

[18] T. Fritz, M. Griepentrog, W. Mokwa, U. Schnakenberg, *Electrochimica Acta* **2003**, *48*, 3029.

[19] S. Basrour, L. Robert, P. Delobelle, *Mater. Sci. Eng. A* **2000**, *288*, 160.

[20] V. L. Popov, J. Gray, V. L. Popov, *Contact Mechanics and Friction: Physical Principles and Applications*, Springer, Berlin Heidelberg, **2010**.

[21] S. Fujinami, E. Ueda, K. Nakajima, T. Nishi, *J. Polym. Sci. Part B Polym. Phys.* **2019**, *57*, 1279.

[22] F. W. DelRio, C. Jaye, D. A. Fischer, R. F. Cook, *Appl. Phys. Lett.* **2009**, *94*, 131909.

[23] B. V. Derjaguin, V. M. Muller, Yu. P. Toporov, *J. Colloid Interface Sci.* **1975**, *53*, 314.

[24] P. Prokopovich, S. Perni, *Colloids Surf. Physicochem. Eng. Asp.* **2011**, *383*, 95.

[25] F. H. Allen, O. Kennard, D. G. Watson, L. Brammer, A. G. Orpen, R. Taylor, *J. Chem. Soc. Perkin Trans.* *2* **1987**, S1.
